# Mast cell–derived factor XIIIA contributes to sexual dimorphic defense against group B streptococcal infections

**DOI:** 10.1172/JCI157999

**Published:** 2022-10-17

**Authors:** Adrian M. Piliponsky, Kavita Sharma, Phoenicia Quach, Alyssa Brokaw, Shayla Nguyen, Austyn Orvis, Siddhartha S. Saha, Nyssa Becker Samanas, Ravin Seepersaud, Yu Ping Tang, Emily Mackey, Gauri Bhise, Claire Gendrin, Anna Furuta, Albert J. Seo, Eric Guga, Irina Miralda, Michelle Coleman, Erin L. Sweeney, Charlotte A. Bäuml, Diana Imhof, Jessica M. Snyder, Adam J. Moeser, Lakshmi Rajagopal

**Affiliations:** 1 Center for Immunity and Immunotherapies, Seattle Children’s Research Institute, Seattle, Washington, USA.; 2 Department of Pediatrics,; 3 Department of Pathology, and; 4 Department of Global Health, University of Washington, Seattle, Washington, USA.; 5 Center for Global Infectious Disease Research, Seattle Children’s Research Institute, Seattle, Washington, USA.; 6 Departments of Large Animal Clinical Sciences, Physiology, Neuroscience Program, and College of Veterinary Medicine, Michigan State University, East Lansing, Michigan, USA.; 7 Pharmaceutical Biochemistry and Bioanalytics, Pharmaceutical Institute, University of Bonn, Germany.; 8 Department of Comparative Medicine, University of Washington, Seattle, Washington, USA.

**Keywords:** Cell Biology, Infectious disease, Bacterial infections, Coagulation, Mast cells

## Abstract

Invasive bacterial infections remain a major cause of human morbidity. Group B streptococcus (GBS) are Gram-positive bacteria that cause invasive infections in humans. Here, we show that factor XIIIA–deficient (FXIIIA-deficient) female mice exhibited significantly increased susceptibility to GBS infections. Additionally, female WT mice had increased levels of FXIIIA and were more resistant to GBS infection compared with isogenic male mice. We observed that administration of exogenous FXIIIA to male mice increased host resistance to GBS infection. Conversely, administration of a FXIIIA transglutaminase inhibitor to female mice decreased host resistance to GBS infection. Interestingly, male gonadectomized mice exhibited decreased sensitivity to GBS infection, suggesting a role for gonadal androgens in host susceptibility. FXIIIA promoted GBS entrapment within fibrin clots by crosslinking fibronectin with ScpB, a fibronectin-binding GBS surface protein. Thus, ScpB-deficient GBS exhibited decreased entrapment within fibrin clots in vitro and increased dissemination during systemic infections. Finally, using mice in which FXIIIA expression was depleted in mast cells, we observed that mast cell–derived FXIIIA contributes to host defense against GBS infection. Our studies provide insights into the effects of sexual dimorphism and mast cells on FXIIIA expression and its interactions with GBS adhesins that mediate bacterial dissemination and pathogenesis.

## Graphical abstract

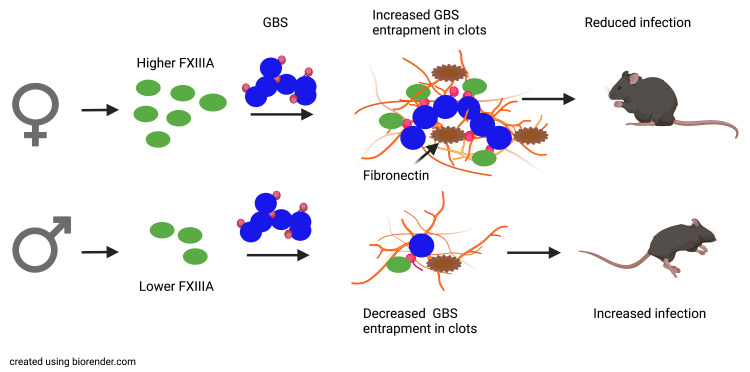

## Introduction

Invasive bacterial infections are one of the leading causes of human morbidity and mortality. An important cause of invasive infections is group B streptococcus (GBS) or *Streptococcus agalactiae*. GBS are Gram-positive bacteria that are frequently associated with preterm births, stillbirths, and infections in neonates and adults (1–8). Annually, at least 3.5 million preterm births, 14,5000 stillbirths, and 319,000 neonatal infections have been attributed to GBS (3). GBS infections have also been described in adults, including the elderly, immunocompromised, diabetic, and even normal, healthy adults (5–10). Together, these indicate the importance of GBS as a clinical pathogen and its ability to cause disease in humans.

Despite its importance as a human pathogen, host factors critical for curtailing GBS infections are not fully understood. Coagulation is an ancestral serine protease cascade (11) that has coevolved with innate immunity (12) and is important for the early innate immune response against invading pathogens (13, 14). To our knowledge, the role of coagulation cascade components in the innate immune response against GBS is unknown. We previously showed that mast cells, which are resident innate immune cells, are important for defense against systemic GBS infections (15, 16). Further, a recent proteomic study revealed that one of the most abundant proteins contained in bone marrow–derived cultured mast cell (BMCMC) granules was a coagulation factor known as factor XIIIA (FXIIIA) (17). Therefore, in this study, we aimed to define the role of FXIIIA in response to GBS infections.

FXIII is an essential component of the coagulation cascade and is a transglutaminase that catalyzes the crosslinking of fibrin to stabilize the fibrin clot (18–20). FXIII circulates in plasma as a tetrameric zymogen consisting of 2 catalytic A subunits (FXIIIA, f13a1) and 2 noncatalytic B subunits (FXIIIB, f13b) (21, 22). In the presence of fibrin, thrombin cleaves FXIII to release the FXIII activation peptides. Subsequently, in the presence of Ca^2+^ and fibrin, FXIIIB dissociates from the complex and FXIIIA is fully activated. Active FXIII (FXIIIA) has transglutaminase activity that catalyzes the formation of ε-(γ-glutamyl) lysine bridges between reactive glutamine and the ε-amino group of a reactive lysine residue (23, 24), which stabilizes the fibrin clot during coagulation. This stabilization is achieved by the formation of crosslinks between fibrin monomers and proteins with antifibrinolytic activity to fibrin (25). Many substrates have been described for FXIIIA, including fibrinogen, fibronectin, and vitronectin (20, 25). FXIIIA has well-known roles in hemostasis, angiogenesis, wound healing, bone metabolism, cardioprotection, and pregnancy (18, 19). In humans, FXIIIA deficiency is characterized by hypocoagulability, leading to excessive bleeding and recurrent miscarriages, which can be successfully managed by exogenous administration of plasma FXIII (26, 27). Of note, a FXIII Val34Leu mutation, found in humans, affects the structure of the crosslinked fibrin clot, ultimately decreasing clot stability (28, 29).

To understand the role of FXIIIA in the innate immune response against GBS, we examined the impact of FXIIIA deficiency and the importance of mast cell–derived FXIIIA in the severity of GBS infections. Our results show that FXIIIA is important for defense against GBS infection. Interestingly, we observed substantial differences in FXIIIA production and GBS dissemination between female and male mice, suggesting a potential sex-dependent mechanism during GBS infection. We further demonstrate that interactions between FXIIIA and the fibronectin-binding bacterial surface protein ScpB promote GBS entrapment into fibrin clots, which likely diminishes GBS dissemination. Finally, we show that mast cell–derived FXIIIA is critical for host defense against GBS infection. These observations provide what we believe are novel insights into sexual dimorphic expression of FXIII and the role of mast cell–derived FXIIIA in GBS infection and pathogenesis.

## Results

### FXIIIA limits bacterial dissemination during systemic GBS infection.

**Figure 1:**
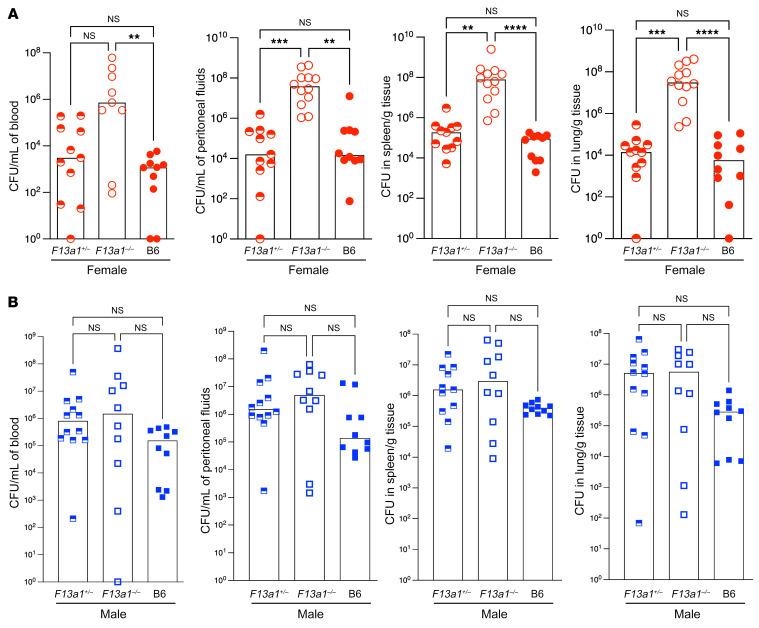
FXIIIA-deficient female mice exhibit increased GBS dissemination during systemic infection. Female FXIIIA-sufficient (B6, *F13a1^+/–^*) and FXIIIA-deficient (*F13a1^–/–^*) mice (**A**) and male FXIIIA-sufficient (B6, *F13a1^+/–^*) and FXIIIA-deficient (*F13a1^–/–^*) mice (**B**) were infected i.p. with 0.5 to 1 × 10^8^ CFU of WT GBS strain A909. *n* = 10–12 mice/group. At 24 hours after GBS infection, bacterial burden was evaluated in blood, peritoneal fluids, spleen, and lungs. Data are represented as medians with values from individual mice depicted. The Kruskal-Wallis test with Dunn’s multiple-comparison test was used for comparison of bacterial burden between FXIIIA-sufficient and FXIIIA-deficient mice. **P < 0.01; ***P < 0.001; **** *P* <0.0001.

Our previous work suggested that innate immune cells, such as mast cells, limit systemic GBS infection (15, 16). As FXIIIA was shown to be released by immune cells, including mast cells and myeloid cells (17, 30), we first investigated whether FXIIIA can affect the outcome of systemic GBS infections. To this end, we inoculated female and male FXIIIA-deficient mice (*F13a1^–/–^*) and control FXIIIA-sufficient mice (*F13a1^+/–^* or C57BL/6 [B6]) using the GBS systemic infection model described previously (15, 16). Mice were inoculated i.p. with 0.5 to 1 × 10^8^ CFU of 1 of 2 clinically relevant WT GBS strains, namely the serotype Ia strain A909 (Figure 1, A and B) or the serotype III strain COH1 (Supplemental Figure 1; supplemental material available online with this article; https://doi.org/10.1172/JCI157999DS1). At 24 hours after GBS infection, mice were euthanized, and the spleen, lungs, blood, and peritoneal fluids were collected to assess GBS dissemination. Tissues were homogenized and enumerated for GBS CFU using methods previously described (15, 16). With both GBS strains, significantly higher bacterial burden was observed in the blood, peritoneal fluids, spleen, and lungs of female FXIIIA-deficient mice when compared with female FXIIIA-sufficient mice (Figure 1A and Supplemental Figure 1), with many of the FXIIIA-deficient mice exhibiting morbidity symptoms by this time. In contrast, no significant differences in bacterial burden were seen between male FXIIIA-deficient and FXIIIA-sufficient mice (Figure 1B).

**Figure 2:**
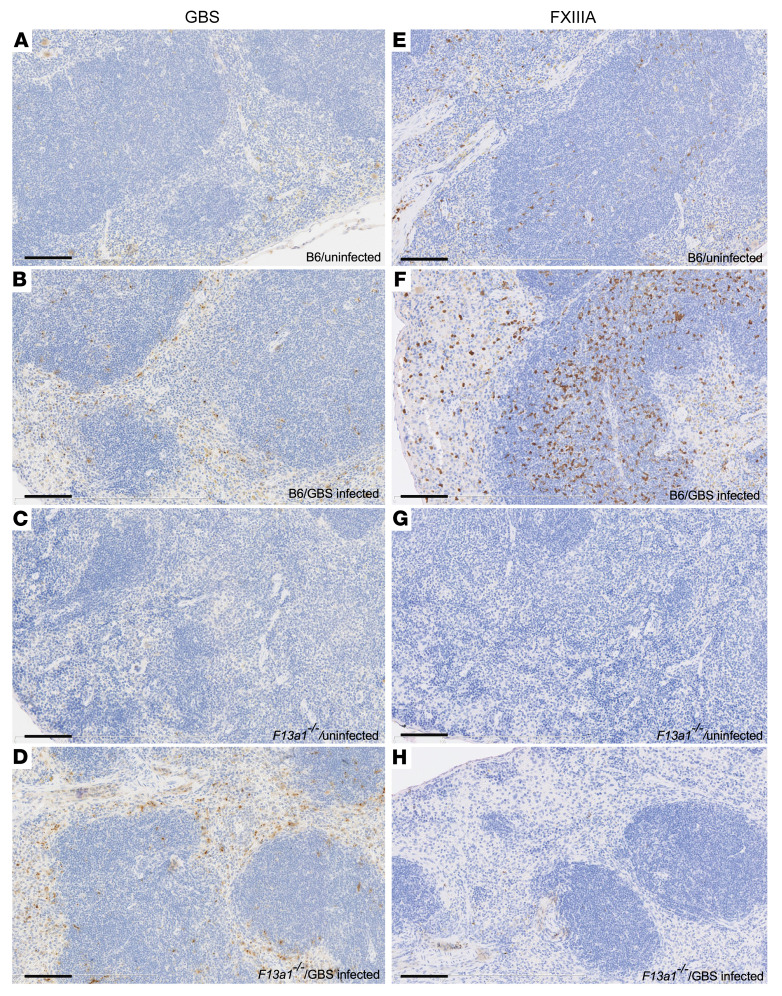
Increased FXIIIA staining in GBS-infected WT mice. Uninfected and GBS-infected mouse spleens from FXIIIA-sufficient (B6) and FXIIIA-deficient mice were immunostained for GBS (left panels) or FXIIIA (right panels). (**A** and **E**) Uninfected B6 mice. (**B** and **F**) GBS-infected B6 mice. (**C** and **G**) Uninfected FXIIIA-deficient mice. (**D** and **H**) GBS-infected FXIIIA-deficient mice. Immunostaining was performed on uninfected FXIIIA-sufficient (*n* = 2) and -deficient mice (*n* = 1) and GBS-infected FXIIIA-sufficient and -deficient mice (*n =* 3/group). GBS and FXIIIA immunopositive cells are shown as brown. Hematoxylin counterstain was used. Original magnification, ×20. Scale bars: 100 μm.

Immunohistochemistry was performed on sections of mouse spleens using GBS and FXIIIA antibodies. With the GBS antibody, minimal nonspecific staining was seen in the spleens from uninfected FXIIIA-sufficient (B6 mice; Figure 2A) or FXIIIA-deficient mice (Figure 2C). In GBS-infected FXIIIA-sufficient (Figure 2B) and FXIIIA-deficient mice (Figure 2D), immunopositive staining for GBS was predominantly seen at the periphery of the lymphoid follicles and in the red pulp, and these appeared more pronounced in the GBS-infected FXIIIA-deficient mice (Figure 2D). As expected, there were minimal to no FXIIIA-positive cells in the spleens of FXIIIA-deficient mice (Figure 2, G and H). While some FXIIIA-positive cells were seen in the spleens of uninfected FXIIIA-sufficient mice (Figure 2E), numerous and strongly intense FXIIIA-immunopositive cells were seen in GBS-infected FXIIIA-sufficient mice (Figure 2F).

We also performed flow cytometry on GBS-infected tissues to determine whether the increased bacterial dissemination observed in female FXIIIA-deficient mice correlated with decreased recruitment of neutrophils and/or macrophages. The results shown in Supplemental Figure 2 indicated no significant difference in recruitment of neutrophils and macrophages in peritoneal fluids, spleen, or lungs between female FXIIIA-deficient mice (*F13a1^–/–^*) and control female FXIIIA-sufficient mice (*F13a1^+/–^*). Similarly, no significant differences in immune cell profiles were observed between uninfected FXIIIA-deficient mice and FXIIIA-sufficient mice (Supplemental Figure 3). Together, these results suggest that FXIIIA limits GBS dissemination during systemic infection in female mice and that this mechanism is independent of neutrophil and macrophage recruitment.

### Sexual dimorphic expression of FXIIIA influences susceptibility to GBS infections.

**Figure 3:**
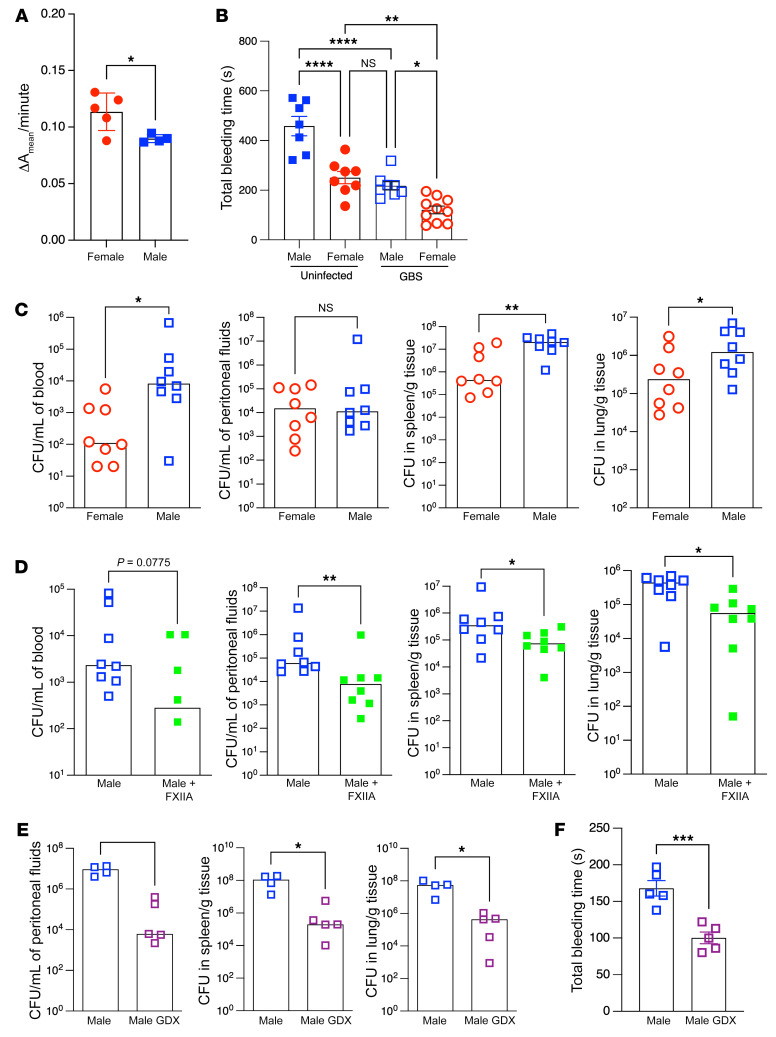
Increased FXIIIA levels promote resistance to GBS infection. (**A**) FXIIIA activity in plasma from uninfected male and female B6 mice (*n* = 4–5/group). Data are represented as mean ± SD with individual values. *P < 0.05, unpaired Student’s *t* test. (**B**) Bleeding time tests on uninfected or GBS-infected B6 male and female mice at 24 hours after infection (*n* = 7–10/group). Data are represented as mean ± SEM with individual values. *P < 0.05; **P < 0.01; ****P < 0.0001, Tukey’s multiple-comparison test following 1-way ANOVA. (**C**) Male and female B6 mice (*n* = 8–9/group) were infected i.p. with 0.5 to 1 × 10^8^ of WT GBS A909. Bacterial burden in blood, peritoneal fluids, spleen, and lungs at 24 hours after infection. Data are represented as median with individual values. *P < 0.05; **P < 0.01, Mann-Whitney *U* test. (**D**) Exogenous FXIIIA (1 unit/mouse) or control PBS was administered i.v. to male B6 mice (*n* = 8 mice/group) 2 hours after GBS infection as above. Bacterial burden in blood, peritoneal fluids, spleen, and lungs at 24 hours after infection. Data are represented as medians with individual values. *P < 0.05; **P < 0.01, Mann-Whitney *U* test. (**E**) GDX and control (sham operated) male B6 mice (*n* = 4-5/group) were infected with WT GBS, and bacterial burden was evaluated in the peritoneal fluids, spleen, and lungs as above. Data are represented as median with individual values. *P < 0.05, Mann-Whitney *U* test. Many of the infected mice in the control group were morbid and hence blood could not be collected. (**F**) Bleeding time tests were performed on GBS-infected male GDX or control (sham operated) B6 mice at 24 hours after infection (*n* = 5/group). Data are represented as mean ± SEM with individual mouse values. ***P < 0.001, unpaired *t* test.

As sexual dimorphism has been shown to affect processes important for host defense against infections (31), we wondered whether male and female WT (B6) mice exhibit differences in FXIIIA activity levels and whether this contributes to differences in severity of GBS infections. To this end, we first compared FXIIIA activity in plasma between male and female WT mice. FXIIIA activity was significantly higher in plasma of female mice when compared with that of isogenic male mice (Figure 3A). As active FXIIIA can be linked to reduced bleeding times (32), we performed bleeding time tests on uninfected male and female WT mice. The results shown in Figure 3B indicate longer bleeding times in uninfected male mice compared with female mice, further confirming intrinsically lower FXIIIA activity in male mice. To evaluate susceptibility to GBS infection, female and male B6 mice were infected i.p. with 0.5 to 1 × 10^8^ CFU of WT GBS. At approximately 24 hours after infection, 1 set of infected mice were euthanized for estimation of bacterial burden and bleeding time tests were performed on the remaining mice to evaluate FXIIIA activity. Interestingly, GBS infection reduced bleeding times in both male and female mice (Figure 3B), indicating activation of the coagulation cascade. Furthermore, GBS-infected female mice exhibited significantly reduced bleeding times compared with GBS-infected male mice (Figure 3B). Bacterial burden was also significantly lower in the blood, spleen, and lungs of GBS-infected female mice compared with male mice (Figure 3C). Together, these data suggest that female mice are more protected than male mice against systemic GBS infection, potentially due to intrinsically higher FXIIIA activity.

We next hypothesized that exogenous administration of FXIIIA to WT male mice may provide the necessary amounts of FXIIIA to diminish GBS systemic infection. Previous studies have shown that human plasma FXIIIA is well tolerated and fully active in mice (32). To test the above hypothesis, male WT mice received exogenous FXIIIA (1 unit/mouse) i.v. 2 hours after GBS infection. Control male mice received PBS i.v. 2 hours after GBS infection. Approximately 24 hours after GBS infection, all groups of mice were euthanized, and organs were harvested for enumeration of GBS CFU as described earlier. Mice that received FXIIIA had significantly decreased bacterial burden in the peritoneal fluids, spleen, and lungs (with trends for lower CFU in blood) when compared with PBS controls (Figure 3D). Thus, exogenous administration of FXIIIA decreased GBS dissemination during infection.

Our observations of differences in FXIIIA activity between male and female mice and sexually dimorphic susceptibility to systemic GBS infections prompted us to determine whether gonadal androgens present in male mice contribute to these phenotypes. To test this hypothesis, WT male mice that were either gonadectomized (GDX) or sham operated (control) were infected with GBS for 24 hours. The mice were then euthanized, and bacterial burden in peritoneal fluids, spleen, and lungs was enumerated. The results shown in Figure 3E indicate that, compared with sham-operated controls, male GDX mice exhibited decreased susceptibility to GBS infections. To compare FXIIIA activity, bleeding time tests were performed on GBS-infected male GDX or control mice. The results shown in Figure 3F indicate significantly lower bleeding times, reflecting increased FXIIIA activity in GBS-infected GDX mice compared with isogenic sham-operated controls. In addition, female GDX mice did not exhibit significant differences in GBS dissemination when compared with sham-operated controls (Supplemental Figure 4). Taken together, these results suggest that gonadal androgens present in male mice increase susceptibility to GBS infection.

### FXIIIA mediates bacterial entrapment within fibrin clots.

**Figure 4:**
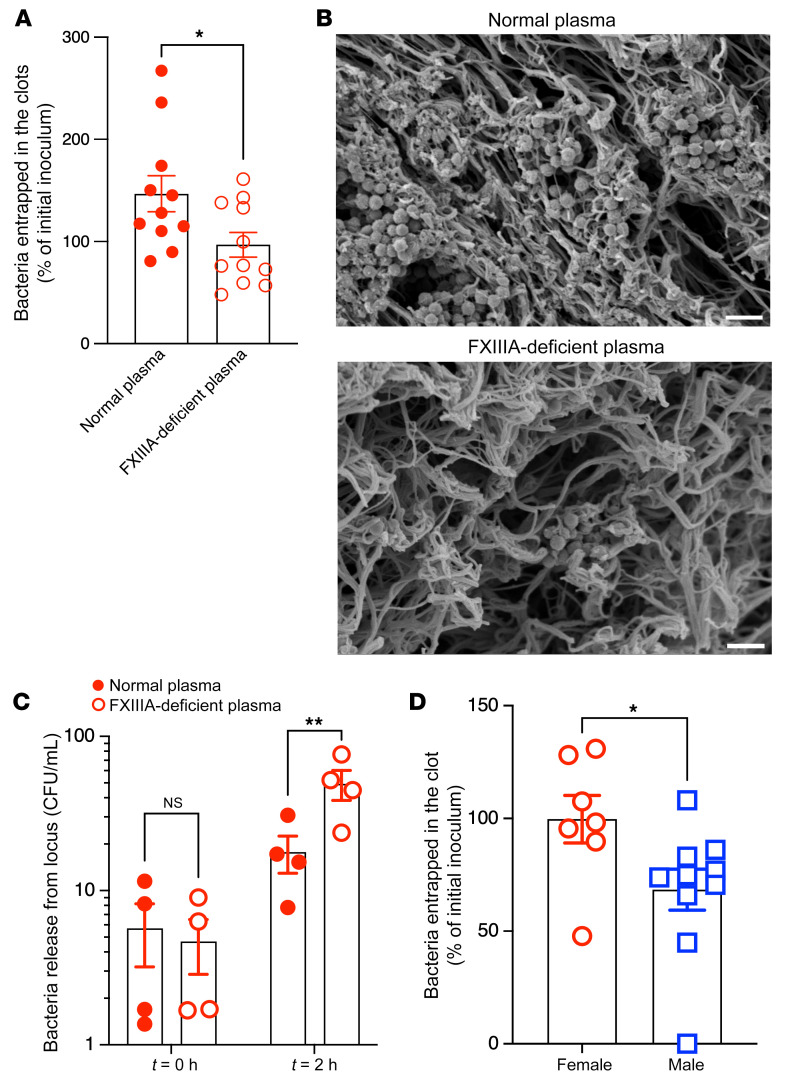
FXIIIA contributes to GBS entrapment within fibrin clots. (**A**–**C**) WT GBS COH1 (10^7^ CFU) in 100 μL was incubated with normal human plasma (Normal plasma) or plasma obtained from patients with congenital FXIIIA deficiency (FXIIIA-deficient plasma). Thrombin was added to induce clotting. (**A**) GBS CFU that were entrapped within the fibrin clot were enumerated. Data are shown as percentage of initial inoculum. *n* > 5. *P <0.05, unpaired *t* test. Data are represented as mean ± SEM. (**B**) Representative scanning electron micrograph showing GBS entrapped in normal plasma or FXIIIA-deficient plasma. Scale bars: 2 μm. (**C**) In separate experiments, clots obtained from normal or FXIIIA-deficient plasma containing entrapped GBS were washed and incubated in buffer at 37°C for 2 hours. At *t* = 0 and 2 hours, the number of bacteria released into the supernatant was determined. Data represent mean ± SEM of 4 independent experiments. **P < 0.01, Šidák’s multiple-comparison test following 2-way ANOVA. (**D**) WT GBS COH1 (10^7^ CFU) was incubated with male or female mouse (B6) plasma. Thrombin was added to induce clotting. GBS CFU that were entrapped within the fibrin clot were enumerated, and data are shown as percentages of initial inoculum. *n =* 7–10. *P <0.05, unpaired *t* test. Data are represented as mean ± SEM.

Previous studies have described a role for human FXIIIA in pathogen entrapment via the clotting system (23, 33, 34). Therefore, we first evaluated whether FXIIIA mediates GBS entrapment within fibrin clots, which can limit bacterial dissemination. To test this possibility, we incubated WT GBS in normal human plasma (normal plasma) or plasma obtained from patients with congenital FXIIIA deficiency (FXIIIA-deficient plasma). After the addition of thrombin to induce clotting, we enumerated GBS CFU that were entrapped within the fibrin clot using serial dilution and plating. We observed significantly fewer GBS CFU in clots generated using FXIIIA-deficient plasma when compared with normal plasma, indicating that FXIIIA significantly enhanced GBS entrapment within the fibrin clot (Figure 4A). Scanning electron microscopy also revealed fewer entrapped GBS in clots generated from FXIIIA-deficient plasma compared with normal plasma (Figure 4B). Additionally, fewer entrapped GBS were released from the clots of normal plasma compared with FXIIIA-deficient plasma (Figure 4C), thereby limiting bacterial dissemination. Furthermore, GBS also exhibited reduced survival in thrombin-activated normal and FXIIIA-deficient human plasma (Supplemental Figure 5), indicating that activation of the coagulation cascade is detrimental for bacterial survival. Consistent with the increased FXIIIA activity observed in female mice (Figure 3), we observed that more GBS were entrapped in clots generated from female mouse plasma when compared with male mouse plasma (Figure 4D). Taken together, these data indicate that FXIIIA contributes to pathogen entrapment in fibrin clots, which can limit bacterial dissemination.

### FXIIIA transglutaminase activity diminishes GBS infection and mediates bacterial entrapment in a fibronectin-dependent manner.

**Figure 5:**
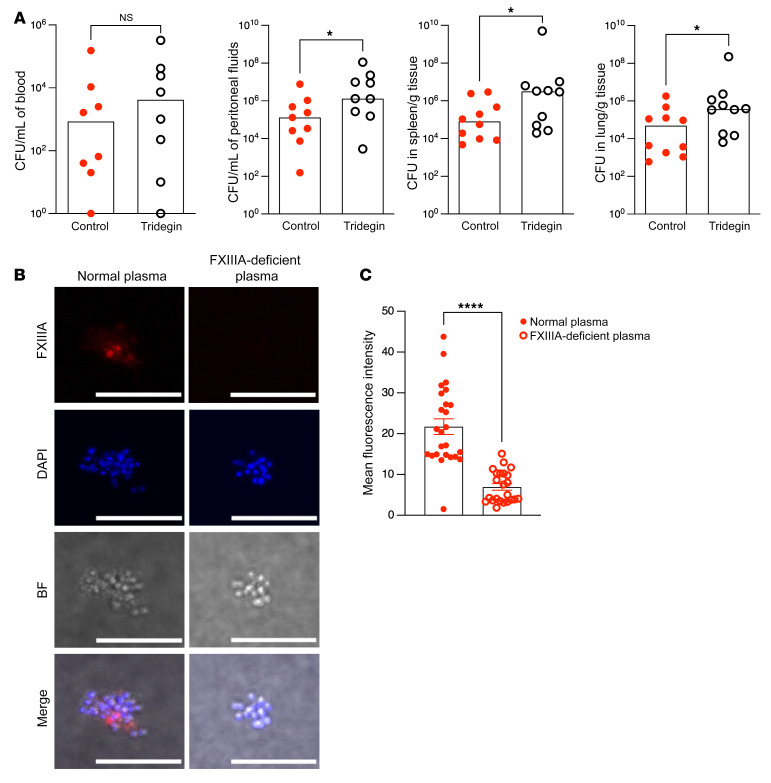
FXIIIA transglutaminase activity is important for limited GBS dissemination. (**A**) Tridegin (6 μg/mouse) or equivalent volume of control PBS was administered i.v. to female B6 mice (*n* = 9–10/group) 2 hours after GBS infection (i.p., WT A909). Bacterial burden was evaluated 24 hours after infection. Data are represented as median with values from individual mice shown. *P < 0.05, Mann-Whitney *U* test. (**B** and **C**) WT GBS COH1 was incubated with normal plasma or FXIIIA-deficient plasma. (**B**) Biotin-cadaverine and streptavidin-cy3 were used to visualize FXIIIA transglutaminase activity on the bacterial surface by immunofluorescence/confocal microscopy. DAPI and bright-field images are also shown. Scale bars: 10 μm. (**C**) Quantification of FXIIIA transglutaminase activity via immunostaining fluorescence intensity. ****P < 0.0001, unpaired *t* test. Data are represented as mean ± SEM.

To determine the importance of FXIIIA transglutaminase activity in GBS infection, we determined whether administration of tridegin (a peptide inhibitor of FXIIIA transglutaminase activity, ref. 35) influences GBS dissemination in infected mice. To this end, tridegin was administered to WT female mice i.v. (6 μg/mouse, retroorbital injection) 2 hours after GBS infection (i.p. with 0.5 to 1 × 10^8^ of WT GBS). Control mice received PBS i.v. at the same time point. At 24 hours after infection, mice were euthanized and organs harvested for enumeration of GBS CFU as above. The results shown in Figure 5A indicate that bacterial burden was significantly increased in the peritoneal fluids, spleen, and lungs of mice that received tridegin when compared with PBS controls. Thus, pharmacological inhibition of FXIIIA transglutaminase activity by tridegin increases the severity of GBS infection.

We next performed fluorescence microscopy to visualize FXIIIA transglutaminase activity on the GBS microbial surface. Previous studies have shown that substrates with small primary amines, such as biotin-cadaverine, can mimic lysine during FXIIIA transglutaminase-mediated crosslinking and mark proteins involved in crosslinking by FXIIIA (23). Thus, GBS were incubated with either normal or FXIIIA-deficient plasma in the presence of thrombin, and biotin-cadaverine was used to detect FXIIIA transglutaminase-mediated crosslinking on the microbial (GBS) cell surface. The results shown in Figure 5, B and C (normal and FXIIIA-deficient human plasma), and Supplemental Figure 6 (mouse WT and FXIIIA-deficient [*F13a1*^–/–^] plasma) indicate increased incorporation of biotin-cadaverine on the surface of the GBS-fibrin complex, with normal/WT plasma compared with FXIIIA-deficient plasma. These data confirm the importance of FXIIIA transglutaminase activity in interactions with the GBS cell surface.

**Figure 6:**
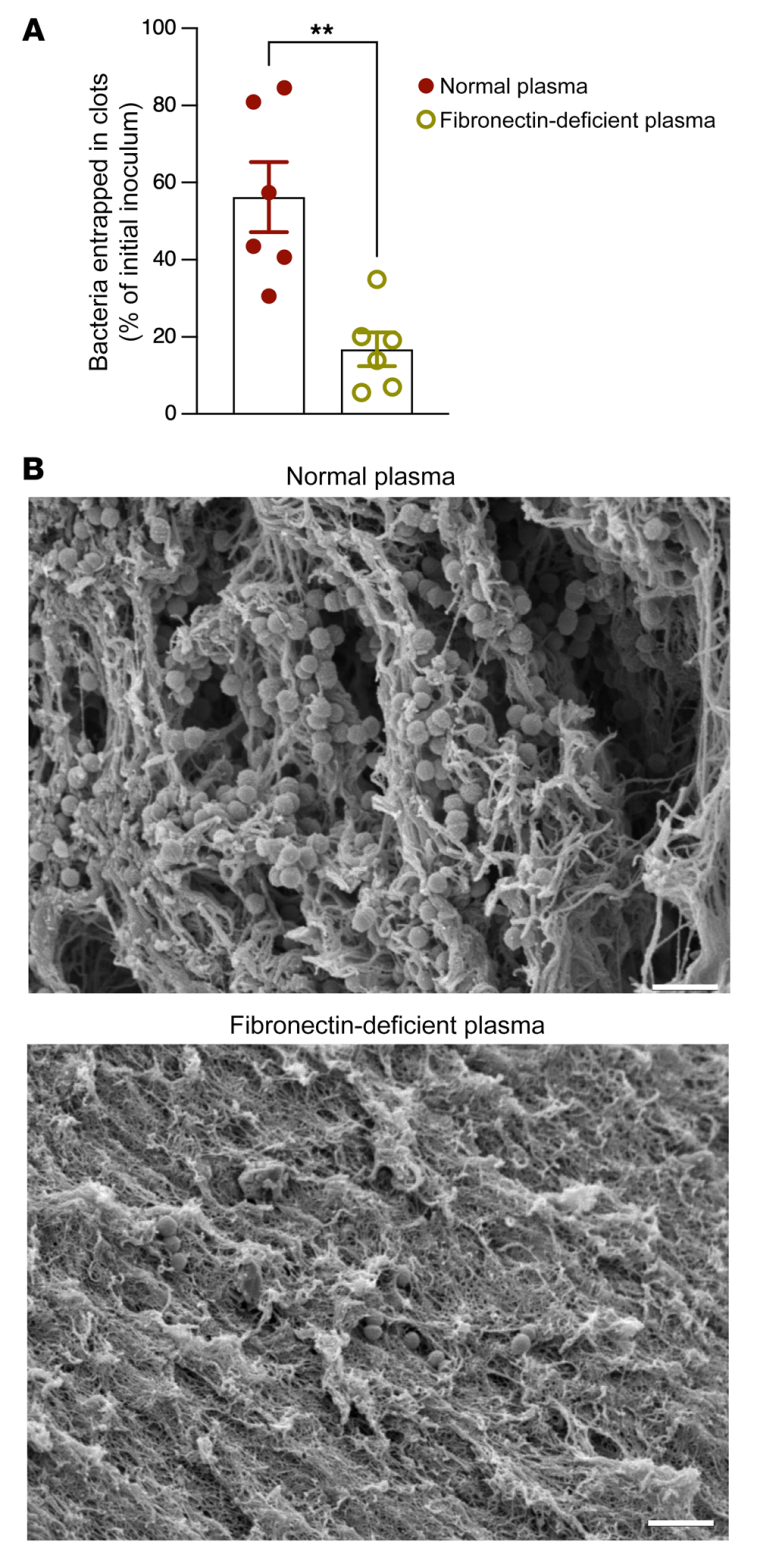
Fibronectin is important for GBS entrapment in fibrin clots. (**A** and **B**) GBS WT COH1 (10^7^ CFU) was incubated in 100 μL of normal or fibronectin-deficient human plasma. (**A**) GBS CFU that were entrapped within the clot were enumerated. Data are shown as percentage of initial inoculum. *n* = 6. **P < 0.01, unpaired *t* test. Data are represented as mean ± SEM. (**B**) Representative scanning electron micrograph showing GBS entrapped in normal plasma or fibronectin-deficient plasma. Scale bar: 2 μm.

As FXIIIA catalyzes crosslinking of soluble fibronectin to the fibrin clot (36), we examined the role of fibronectin in GBS entrapment within fibrin clots. To this end, WT GBS was incubated in normal human plasma (normal plasma) or human plasma deficient in fibronectin. The results shown in Figure 6A indicate that significantly fewer GBS CFU were entrapped in clots generated using fibronectin-deficient plasma when compared with normal human plasma. Furthermore, scanning electron microscopy revealed substantially altered clot morphology and fewer entrapped GBS in fibronectin-deficient plasma when compared with normal human plasma (Figure 6B). Taken together, these results indicate that both FXIIIA and fibronectin are important for GBS entrapment within the fibrin clot.

### The fibronectin-binding protein ScpB promotes bacterial entrapment and limits GBS dissemination.

**Figure 7:**
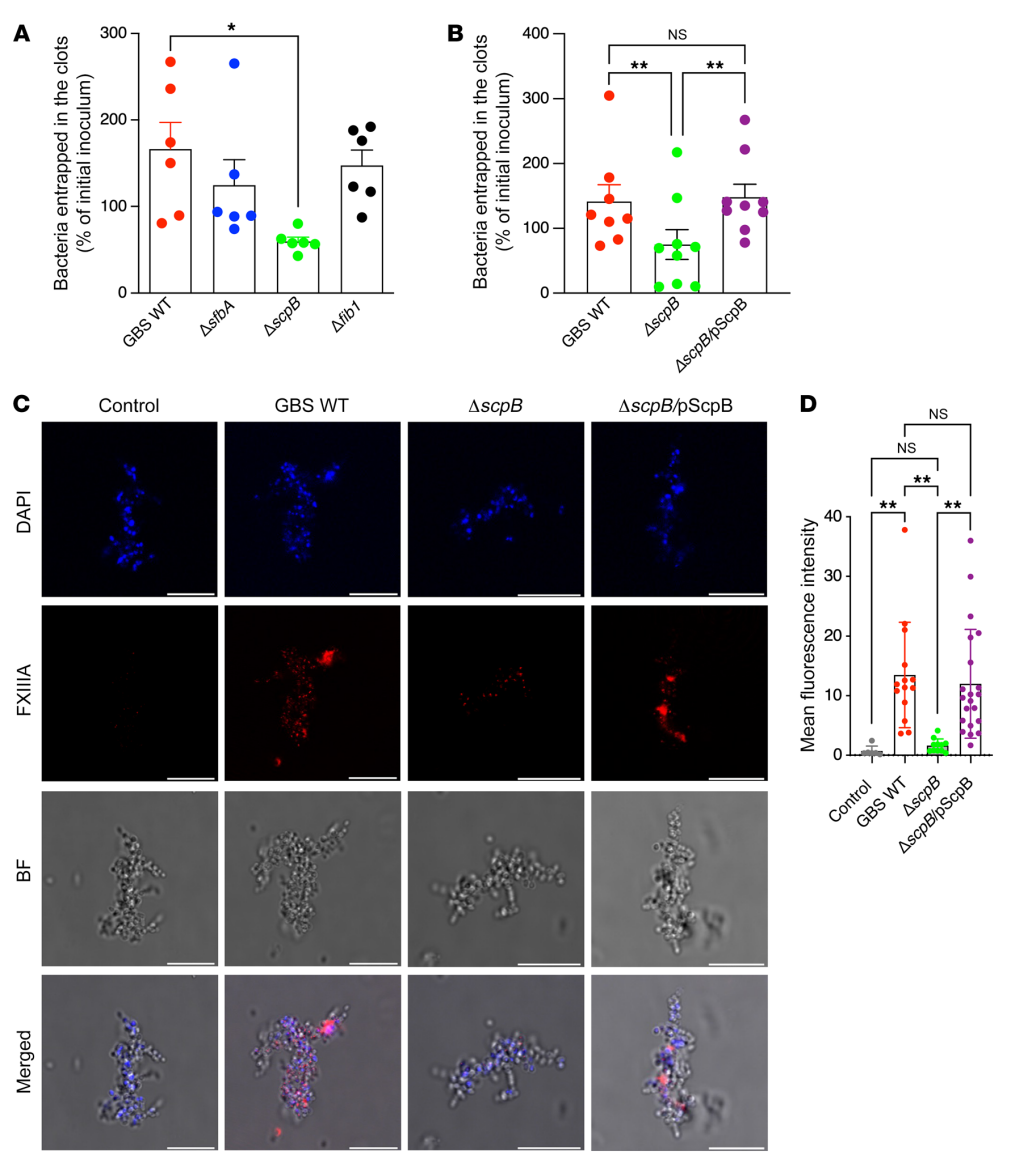
The fibronectin-binding protein ScpB promotes GBS entrapment within fibrin clots. (**A**) Approximately 10^7^ CFU of GBS WT strain COH1 or isogenic mutants deficient in expression of fibronectin-binding proteins SfbA, ScpB, and FibA (denoted as Δ*sfbA*, Δ*scpB*, Δ*fibA*, respectively) were incubated in normal human plasma. After the addition of thrombin to induce clotting, GBS CFU that were entrapped within the fibrin clot were enumerated. Data are shown as percentage of initial inoculum. *n* = 6. *P < 0.05, Tukey’s multiple-comparison test following 1-way ANOVA. Data are represented as mean ± SEM. (**B**) Complementation of Δ*scpB* with the WT allele exhibited increased entrapment in fibrin clots. GBS WT COH1 and isogenic Δ*scpB* and the complemented strain Δ*scpB/*pScpB were incubated in normal human plasma. After the addition of thrombin to induce clotting, GBS CFU that were entrapped within the fibrin clot were enumerated. Data are shown as percentage of initial inoculum. *n* ≥ 8. **P < 0.01, Tukey’s multiple-comparison test following 1-way ANOVA. Data are represented as mean ± SEM. (**C**) Plasma obtained from WT mice was activated with thrombin in the presence of either GBS WT COH1, isogenic Δ*scpB*, or the complemented strain Δ*scpB/*pScpB. Biotin-cadaverine and streptavidin-cy3 were used to visualize FXIII transglutaminase activity at the bacterial surface using immunofluorescence/confocal microscopy. DAPI and bright field images are also shown. Control samples were processed as above, except that biotin-cadaverine was not added to these samples. Scale bars: 10 μm. (**D**) Quantification of FXIIIA transglutaminase activity via immunostaining fluorescence intensity. **P < 0.01, Šidák’s multiple-comparison test following 1-way ANOVA. Data are represented as mean ± SD.

To gain further insight into FXIIIA and fibronectin interactions with GBS, we determined whether a GBS surface protein could promote bacterial entrapment in the fibrin clots. A fibronectin-binding protein of *Staphylococcus aureus* was suggested to act as a substrate for FXIIIA (24), prompting us to determine whether GBS fibronectin-binding proteins served a similar role. To this end, we compared bacterial entrapment in fibrin clots between GBS WT and isogenic mutants lacking known fibronectin-binding proteins, such as ScpB (37), SfbA (38), and Fib1 (39). GBS deficient in the expression of the fibronectin-binding protein ScpB exhibited significantly reduced entrapment within fibrin clots when compared with WT; this was not observed with GBS deficient in other fibronectin-binding proteins, such as SfbA or Fib1 (Figure 7A). Additionally, complementation of Δ*scpB* with the WT *scpB* allele restored GBS entrapment within fibrin clots to levels comparable to those seen with GBS WT (Figure 7B). Fluorescence microscopy was also used to visualize FXIIIA transglutaminase activity on the GBS microbial surface. Consistent with previous observations, we noted that FXIIIA-mediated incorporation of biotin-cadaverine on the GBS bacterial surface was ScpB dependent (Figure 7, C and D). Of note, bacterial growth and colony morphology were not markedly different between the ScpB-proficient and -deficient GBS strains (Supplemental Figure 7).

**Figure 8:**
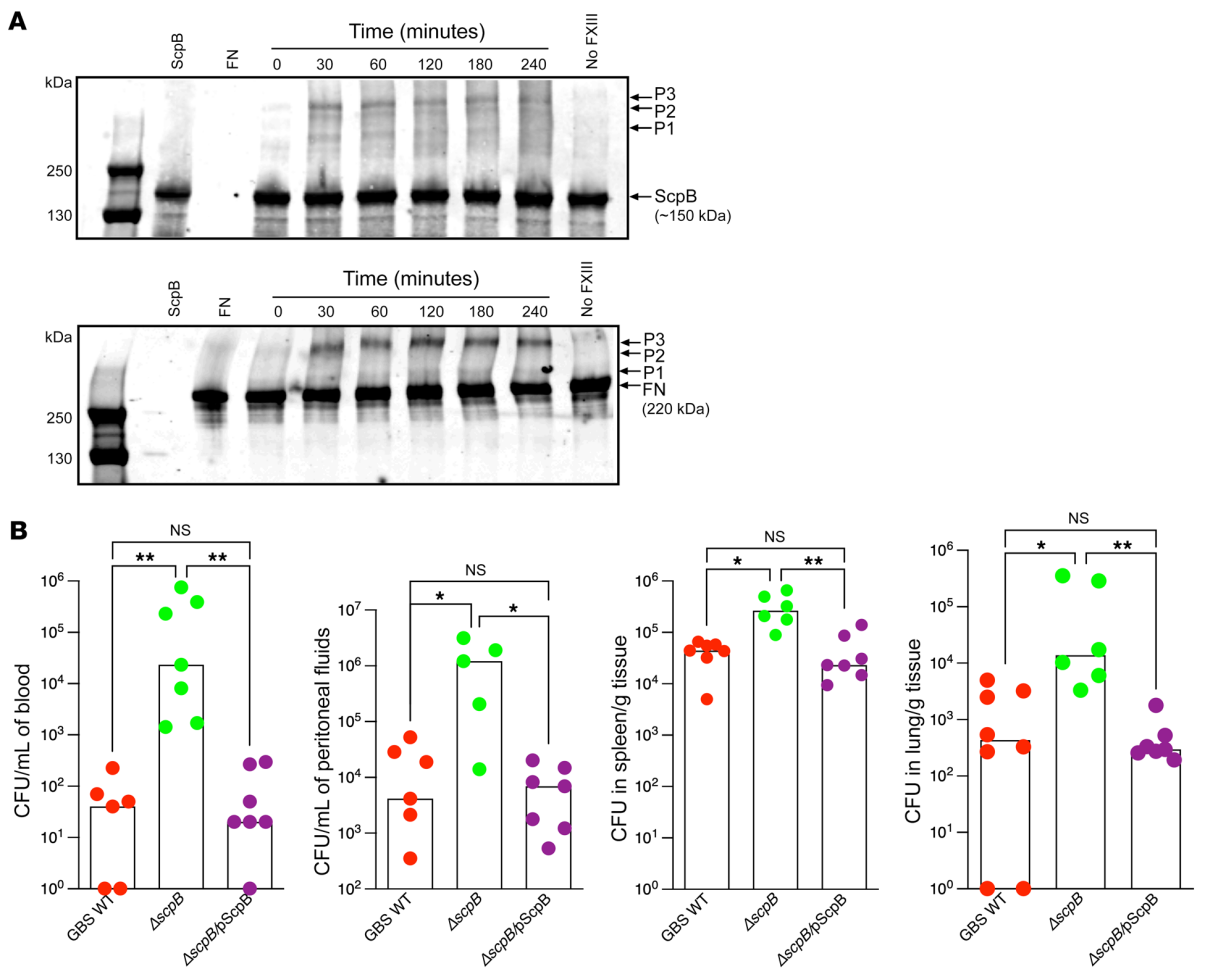
GBS ScpB forms protein complexes with fibronectin and attenuates GBS dissemination. (**A**) FXIIIA-mediated crosslinking between purified ScpB and fibronectin. ScpB (2 μM) was incubated with fibronectin (1 μM) and FXIIIA (30 μg/mL) in TBS, pH 7.4, 5 mM CaCl_2_ buffer at 25°C from 0 to 240 minutes. Aliquots were removed at the indicated times and were separated on SDS-PAGE, followed by Western blotting with either the anti-ScpB (top panel) or anti-fibronectin (bottom panel) antibodies. Controls included ScpB alone, fibronectin (FN) alone, or samples lacking FXIIIA. Arrows indicate the positions of the ScpB, FN, and products of the crosslinking reaction, which are depicted as P1, P2, and P3. A representative blot from 3 independent experiments is shown. (**B**) WT B6 mice were infected i.p. with 0.5 to 1 × 10^8^ CFU of WT GBS strain COH1, isogenic ScpB-deficient GBS (Δ*scpB*), or the complemented strain(Δ*scpB/*pScpB). At 24 hours after infection, bacterial burden was evaluated in blood, peritoneal fluids, spleen, and lungs. Data are shown as medians with boxes representing values from individual mice. *P < 0.05; **P < 0.01, Kruskal-Wallis test with Dunn’s multiple-comparison test.

Previous studies showed that FXIIIA transglutaminase activity promoted the formation of covalently crosslinked heteropolymers between fibronectin and a staphylococcal fibronectin-binding protein (24). To determine whether transglutaminase activity of FXIIIA stabilizes a protein complex between GBS ScpB and fibronectin and induces the formation of heteropolymers, we incubated recombinant ScpB with fibronectin and FXIIIA and probed for ScpB-fibronectin complexes by Western blotting. The results shown in Figure 8A indicate that ScpB and fibronectin undergo crosslinking, observed as higher molecular mass species detected by both anti-ScpB and anti-fibronectin antibodies (see P1–P3 in top and bottom panels). Formation of these polymers required FXIIIA, as they were not detected in reactions lacking FXIIIA (Supplemental Figure 8). In the absence of fibronectin, FXIIIA can catalyze the formation of ScpB multimers (Supplemental Figure 8), which likely do not promote bacterial entrapment, as significant GBS entrapment was not seen with fibronectin-deficient human plasma (Figure 6). Taken together, these data suggest that FXIIIA catalyzes the formation of covalently crosslinked heteropolymers comprising ScpB and fibronectin that facilitate GBS entrapment within fibrin clots.

Based on our findings that ScpB promotes GBS entrapment, we hypothesized that GBS deficient in ScpB would exhibit exacerbated dissemination during infection. To this end, WT female mice were infected i.p. with either WT GBS or isogenic Δ*scpB* or the complemented strain Δ*scpB*/pScpB. At 24 hours after infection, the mice were euthanized and bacterial burden in blood, peritoneal fluids, spleen, and lungs was enumerated. As shown in Figure 8B, increased bacterial loads were seen in mice infected with Δ*scpB* when compared with either WT or the complemented ScpB strain. These data provide insight into how the presence of ScpB may diminish the severity of systemic GBS infections.

### The fibronectin-binding PDF region of ScpB mediates bacterial entrapment and limits GBS dissemination.

Previous studies indicated that a phase display fragment (PDF) region in ScpB comprising amino acids 116–227 is important for fibronectin binding (37). To this end, we generated a ScpB variant lacking the PDF region (ScpBΔPDF) and assessed heteropolymer formation with fibronectin. Western blot analysis of ScpBΔPDF after coincubation with fibronectin and FXIIIA revealed that heteropolymers were reduced in abundance (e.g., >460 kDa) compared with that seen with full-length ScpB (Supplemental Figure 9); these results are consistent with previous observations that the PDF domain binds fibronectin with high affinity, but at lower levels compared with full-length ScpB (37), suggesting that other regions of ScpB may also be FXIIIA substrates. In the absence of fibronectin, higher molecular weight species were not seen with the ScpBΔPDF, following coincubation with FXIIIA (Supplemental Figure 9), suggesting that the PDF region is important for FXIIIA crosslinked ScpB homopolymers.

**Figure 9:**
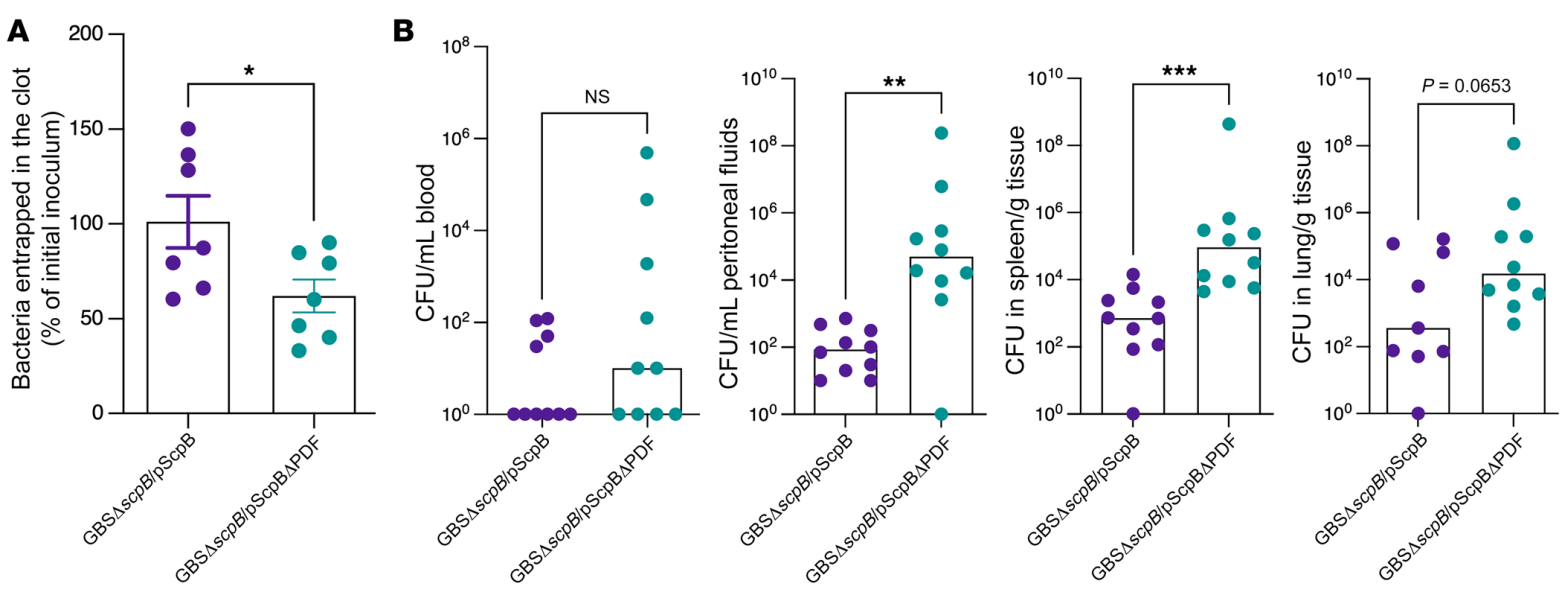
ScpB PDF domain is important for GBS entrapment. (**A**) Approximately 10^7^ CFU of GBS COH1 with and without the PDF domain in ScpB (GBSΔ*scpB/*pScpB and GBSΔ*scpB/*pScpBΔPDF) was incubated in normal human plasma. After the addition of thrombin to induce clotting, GBS CFU that were entrapped within the fibrin clot were enumerated. Data are shown as percentage of initial inoculum. *n* = 7. *P < 0.05, unpaired *t* test. Data are represented as mean ± SEM. (**B**) Female B6 mice were infected i.p. with 0.5 to 1 × 10^8^ CFU of the above GBS strains (*n* =10 mice/group). At 24 hours after GBS infection, bacterial burden was evaluated in blood, peritoneal fluids, spleen, and lungs. Data are shown as medians with circles representing values from individual mice. ** *P* < 0.01; *** *P* < 0.001. Mann-Whitney *U* test was used for comparisons of bacterial burden between groups.

To evaluate the role of the PDF region in FXIIIA-mediated GBS entrapment, we performed coagulation assays using GBS strains with and without the PDF region in ScpB. The results shown in Figure 9A indicate that, when compared with the isogenic GBS strain expressing full-length ScpB (GBSΔ*scpB*/pScpB), the GBS strain lacking the PDF domain in ScpB (GBSΔ*scpB*/pScpBΔPDF) shows fewer entrapped bacteria. Consistent with these observations, significantly increased bacterial dissemination was seen in the peritoneal fluids and spleens (and trends toward higher CFU in lungs) of mice infected with GBS lacking the PDF domain in ScpB (Δ*scpB*/pScpBΔPDF), compared with the isogenic strain expressing full-length ScpB (Δ*scpB*/pScpB) (Figure 9B). Taken together, these data suggest that the PDF domain is important for ScpB-dependent GBS entrapment and diminished dissemination in vivo.

### FXIIIA deficiency in mast cells influences susceptibility to GBS infections.

**Figure 10:**
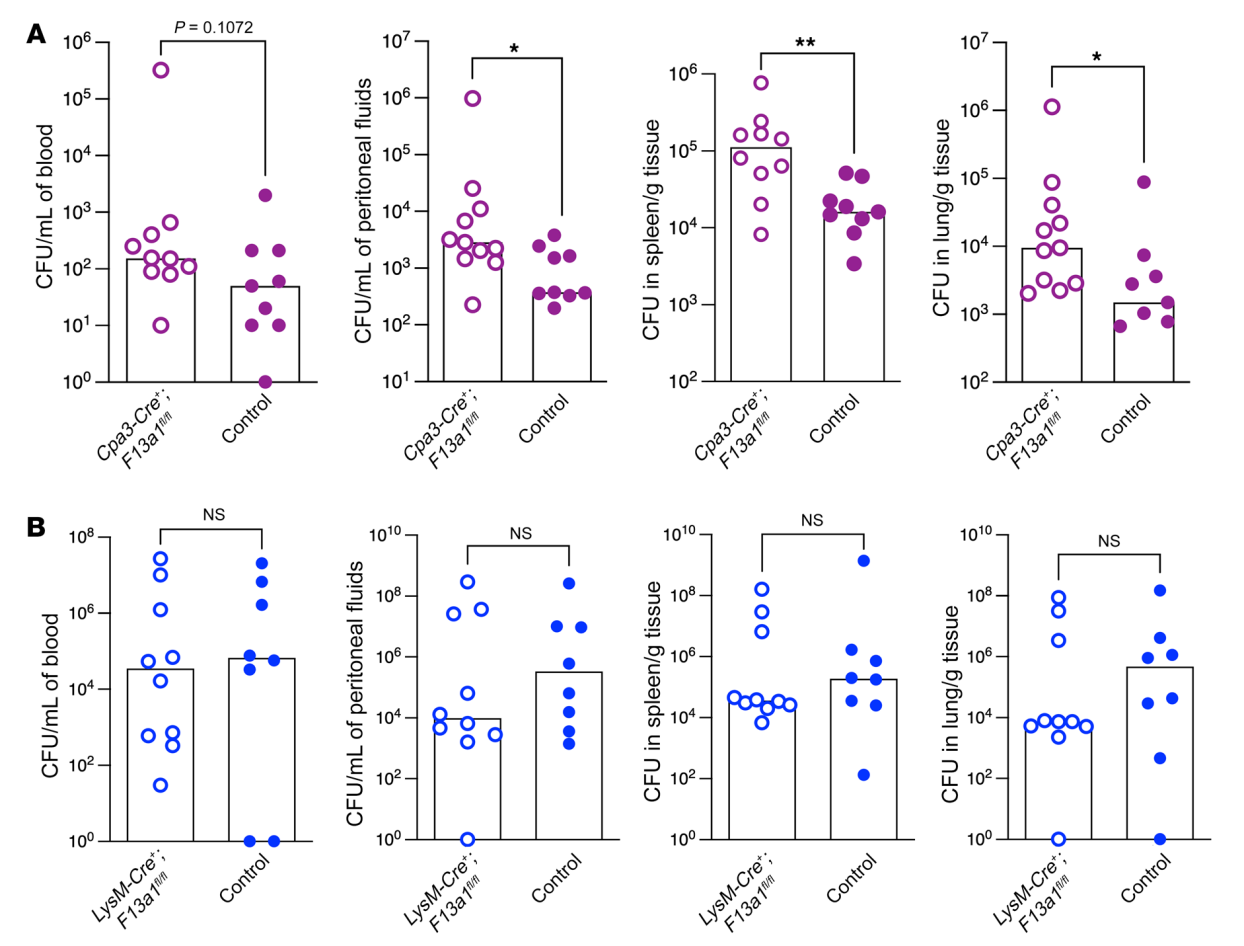
Mast cell–derived FXIIIA is important for defense against GBS infections. (**A**) Female mice with mast cells lacking FXIIIA (*Cpa3-Cre*^+^; *F13a1^fl/fl^*) and control mice (*n* = 9–11/group) were infected i.p. with 0.5 to 1 × 10^8^ of WT GBS strain A909. At 24 hours after infection, bacterial burden was evaluated in peritoneal fluids, spleen, and lungs. Data are shown as medians with values from individual mice also shown. *P < 0.05; **P < 0.01. Mann-Whitney *U* test was used for comparisons of bacterial burden between the GBS strains. (**B**) Female mice with myeloid cells lacking FXIIIA (*LysM-Cre*^+^; *F13a1^fl/fl^*) and control mice were infected with WT GBS as above. At 24 hours after infection, bacterial burden was evaluated in blood, peritoneal fluids, spleen, and lungs. Data are shown as medians with values from individual mice also shown. Mann-Whitney *U* test was used for comparisons of bacterial burden between the GBS strains.

**Figure 11:**
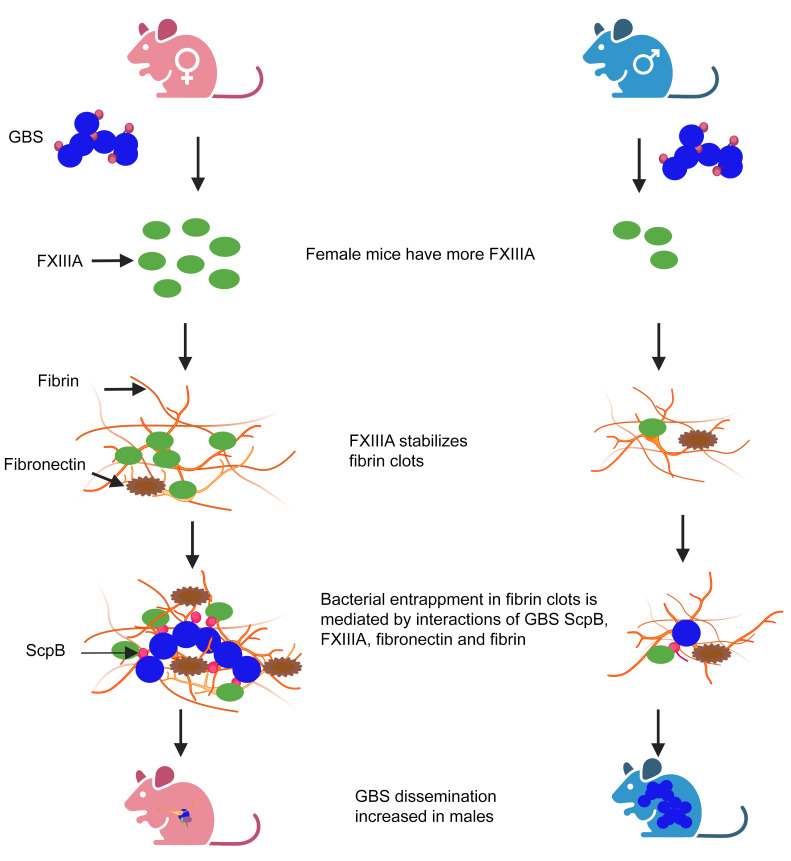
Increased FXIIIA in female mice provides protection against GBS infection. Proposed model shows that female mice produce increased levels of FXIIIA. FXIIIA stabilizes fibrin clots. During GBS infection, FXIIIA interacts with the GBS fibronectin-binding surface protein ScpB and fibronectin, promoting bacterial entrapment, leading to diminished GBS dissemination and infection. Created with BioRender.com.

FXIIIA can be synthesized by a variety of immune cells, including myeloid cells and mast cells (17, 24, 30). We next attempted to discern whether mast cell– and/or myeloid cell–derived FXIIIA is critical for regulation of GBS systemic infections. For this purpose, we generated mice in which FXIIIA expression was depleted in either mast cells or myeloid cells. For depletion of FXIIIA expression in mast cells, a conditional knockout mouse was obtained by crossing *Cpa3-Cre^+^* mice (40) with mice containing *loxP*-flanked *F13a1* alleles (30) (*Cpa3-Cre*^+^;*F13a1^fl/fl^* mice). BMCMCs generated from *Cpa3-Cre*^+^; *F13a1^fl/fl^* mice exhibited significantly reduced FXIIIA levels compared with BMCMCs generated from littermate controls (Supplemental Figure 10). Mice with FXIIIA deficiency in myeloid cells were obtained by crossing *LysM-Cre^+^* mice with those containing *loxP*-flanked *F13a1* alleles (30) (*LysM-Cre*^+^; *F13a1^fl/fl^* mice). Female mice of both these strains were infected i.p. with WT GBS, as described earlier. The results shown in Figure 10A indicate that FXIIIA deficiency in mast cells significantly exacerbated systemic GBS infections compared with that in controls, and this result was not recapitulated in mice with FXIIIA deficiency in myeloid cells (Figure 10B). Furthermore, no significant differences in GBS dissemination were observed between male and female mice lacking FXIIIA in mast cells (Supplemental Figure 11), suggesting that the sexually dimorphic resistance to GBS systemic infection can be attributed to mast cell–derived FXIIIA. Finally, consistent with our results using i.p. inoculation, i.v. inoculation of GBS also significantly increased bacterial burden in female mice with FXIIIA deficiency in mast cells (Supplemental Figure 12). Collectively, our results indicate that sexually dimorphic expression of FXIIIA influences susceptibility to GBS infections, with mast cells being an important source of FXIIIA for host defense (Figure 11).

## Discussion

In this study, we describe an important role for FXIIIA, a critical component of the coagulation cascade, in limiting bacterial dissemination during GBS infections. We observed that mice deficient in FXIIIA exhibited greater susceptibility to systemic GBS infections. Consistent with this observation, we show that administration of exogenous FXIIIA diminished bacterial dissemination, whereas administration of a FXIIIA inhibitor increased GBS dissemination in WT mice. We further showed that FXIIIA catalyzes transglutaminase reactions between fibronectin and the GBS fibronectin-binding surface protein ScpB to promote bacterial entrapment within fibrin clots, which likely limits bacterial dissemination in vivo. Formation of heteropolymers comprising ScpB and fibronectin did not occur in the absence of FXIIIA, and substantially reduced GBS entrapment was seen in plasma lacking FXIIIA or fibronectin. Together, these data suggest an important role for FXIIIA and fibronectin in GBS entrapment within clots.

Consistent with the importance of fibronectin, GBS lacking the fibronectin-binding protein ScpB exhibited enhanced GBS dissemination during systemic GBS infection. Although ScpB was initially characterized as a C5a peptidase with the ability to cleave complement factor C5a (41), subsequent studies have demonstrated that ScpB can also bind fibronectin with high affinity (42). Furthermore, 22% of virulent serotype III isolates with the conserved *scpB* locus lacked C5a peptidase activity (43), suggesting that fibronectin binding by ScpB may play a crucial role in GBS evolution and pathogenesis. A region of ScpB known as PDF was previously shown to bind fibronectin with high affinity (37). In our study, we observed that ScpB lacking the PDF domain exhibited reduced heteropolymer formation with fibronectin and that GBS strains lacking this domain exhibited decreased bacterial entrapment in thrombin-induced clots and increased in vivo dissemination. These data further establish the importance of fibronectin binding for GBS entrapment and bacterial virulence.

While immunization with ScpB was shown to diminish mucosal colonization in young mice (44), we show that loss of ScpB exacerbates GBS pathogenesis, particularly during bloodstream infections. Together, these data suggest that, while the fibronectin-binding activity of ScpB may contribute to certain facets of the GBS disease cycle, such as adherence and colonization, its interaction with fibronectin and FXIIIA in the coagulation cascade may serve to control bacterial dissemination to dampen severe bacteremia and host mortality. Homopolymer formation observed with ScpB in the presence of FXIIIA suggests that crosslinking between GBS cells may also serve to limit GBS dissemination. Collectively, these results highlight diverse roles enacted by bacterial determinants such as ScpB during GBS disease progression and pathogenesis.

Although the role of FXIIIA and fibronectin in the host innate immune response against GBS was previously unappreciated, coagulation, a serine protease cascade, has been described as an important immune response for other invading pathogens. For example, FXIIIA contributed to the containment and elimination of *Streptococcus pyogenes* skin infections in a murine model (29, 30). The importance of fibronectin in bacterial entrapment within thrombin-induced clots was previously also described for *S*. *aureus* (45, 46). However, unlike the role of FXIIIA in containment of GBS and *S*. *pyogenes*, *S*. *aureus* exploits the mammalian coagulation cascade for pathogenesis. Through the combined action of bacterial-encoded coagulases, fibrinogen-binding proteins, and von Willebrand factor binding protein (vWbp) to promote abscess formation, *S*. *aureus* can evade the host immune defenses for subsequent dissemination and pathogenesis (47, 48). In the case of GBS, we report that GBS infection activates the clotting cascade that decreases bacterial survival in thrombin-activated plasma. Fewer GBS are also released from the thrombin-induced clots after entrapment, suggesting that entrapment limits GBS dissemination.

Histological analysis of tissue sections of infected mice revealed increased FXIIIA immunostaining in spleens of GBS-infected WT mice and increased GBS immunostaining in FXIIIA-deficient mice; these observations are consistent with our data on bacterial dissemination in these mouse strains. Although we attempted to identify bacterial entrapment within clots in tissue sections of infected mice using histology, these results were inconclusive and further in-depth studies are needed to enable visualization of bacterial entrapment in vivo. Studies have also described the presence of host leukocytes, such as polymorphonuclear neutrophils (PMNs), in FXIIIA crosslinked fibrin clots; these leukocytes release proteolytic enzymes that inactivate FXIIIA within the clot and restrict clot size to eventually promote clot resolution (49, 50). Together with our findings, these observations suggest that early activation of components of the coagulation cascade may allow the host to restrict bacterial dissemination and promote recruitment of innate immune cells for further pathogen clearance, culminating in clot resolution. Further studies aiming to assess the interaction between components of the coagulation cascade and innate immune defenses during bacterial infections would contribute to a more thorough understanding of how host defenses curtail GBS infections.

While we show that intrinsic FXIIIA levels influence host susceptibility to GBS infections and that activation of FXIIIA limits GBS dissemination, FXII has also been reported to affect pathogenesis of a hypervirulent GBS strain (10). This unique hypervirulent GBS strain (LUMC16) produces remarkably increased levels of hemolytic pigment toxin and was shown to induce blood clotting, wherein FXII activity was detected on the surface of GBS. It is likely that when GBS produces extremely high levels of its hemolytic toxin, this triggers FXII-mediated plasma clotting, leading to toxic shock and *purpura fulminans* as reported (10). However, given that most GBS strains do not express such high levels of the hemolytic pigment as the above (10) and based on our results with 2 different WT GBS strains (A909 and COH1) with modest hemolysin expression typically seen in many clinical isolates, we predict that FXIIIA and fibronectin play a critical role in limiting GBS dissemination during systemic infection. We observed that, owing to intrinsically higher FXIIIA levels, female mice are more resistant to systemic GBS infections when compared with male mice. Previous studies in humans reported a slight but statistically significant increase in FXIIIA and FXIIIB levels in females compared with males (51). While the role of sex on susceptibility to GBS infections is not completely elucidated, GBS colonization was reported to be higher in male infants (52) and GBS infections were also reported to be higher in adult males (53, 54). In a small subset of population-based studies that report sex-specific data for GBS outcomes in infants, higher prevalence of invasive infections in male infants was reported in some cases (55–57). In other studies, male fetuses were suggested to be at greater risk for preterm birth (58). In pregnant rats, placentas of male fetuses exhibited a higher inflammatory response following GBS infection when compared with female fetuses (59), suggesting that males may mount an exaggerated inflammatory response to compensate for deficiencies in effective mechanisms to combat GBS infection. A recent study indicated that boys with previous invasive GBS disease were at higher risk for neurodevelopmental impairments (60). Studies on the effect of male versus female hormones on susceptibility to GBS infections are less clear, with certain studies indicating that vaginal progesterone can decrease GBS colonization at term (61), whereas other studies indicate that estradiol and progesterone can impair the innate immune responses of mononuclear cells in newborns (62) and favor GBS intestinal translocation through neonatal M cells (63). In a more recent study using rats, androgens were described as upregulating the placental innate immune response, thereby contributing to several developmental impairments resulting from perinatal infection/inflammation (64). These observations are intriguing and provide the foundation for future comprehensive studies to determine how sex and gonadal hormones may influence levels of FXIIIA, host innate immunity, and susceptibility of human newborns and adults to bacterial infections.

Sexual dimorphism in mast cells has also been suggested as having direct implications for diseases, including those associated with a female predominance (65, 66). For instance, prevalence of allergic disorders and autoimmunity is known to be higher in females than males (65, 67), and female mast cells exhibit an increased capacity to synthesize and store mediators associated with development of these disorders (68, 69). While the evolutionary advantages for females having mast cells that exhibit increased responsiveness to stimuli in certain conditions is not known, here we explored the role of sexual dimorphism and mast cells based on our previous findings showing that coagulation FXIIIA is abundantly released by activated mast cells (17). Our studies reveal that mast cells are an important source of FXIIIA and that FXIIIA limits GBS dissemination and infection in a fibronectin-dependent manner.

## Methods

### Materials and bacterial strains.

Chemicals used in this study were purchased from Sigma-Aldrich, unless stated otherwise. Commercial antibodies used in this study included the anti-fibronectin antibody (clone FBN11, Invitrogen), anti-FXIIIA antibody (clone ab1834, Abcam), anti-transferrin antibody (clone PA3-913, Thermo Scientific), polyclonal rabbit anti-human fibrinogen antibody (code A0080, Dako), and anti-streptococcus group B antibody (catalog ab53584, Abcam). Tridegin was synthesized in house following procedures previously described (35, 70), and FXIIIA was purchased from Haematologic Technologies.

The WT GBS strains used in this study were clinical isolates obtained from human newborns; these included A909, a capsular serotype Ia strain (71), and COH1, a capsular serotype III hypervirulent ST-17 clone (72). GBS were grown using tryptic soy broth (TSB) or tryptic soy agar (TSA) (Difco Laboratories) at 30°C or 37°C in 5% CO_2_. GBS mutants deficient for fibronectin-binding protein ScpB (Δ*scpB*, ref. 37), SfbA (Δ*sfbA*, ref. 38), and Fib1 (Δ*fib1*, ref. 39) were previously described. The complemented pScpB plasmid was previously constructed (37).

### Activity assay.

FXIIIA activity in plasma was measured using the Technochrom FXIII Kit per the manufacturer’s instructions (Diapharma Group Inc.). Briefly, 25 μL of either male or female mouse plasma treated with sodium citrate (0.01 M final concentration) was mixed with either the sample or blank reagent. Absorbance at 340 nm was measured at 5, 7, 9, and 11 minutes. FXIIIA activity is reported as the average blank-corrected change in absorbance per minute during the linear phase of the curve.

### Mice.

WT male or female B6 mice were obtained from Jackson Laboratory as needed. Factor XIII–deficient mice (*F13a1^–/–^*) and mice with loxP-flanked F13a1 alleles (*F13a1^fl/fl^*) (30, 32) on a B6 background (30) were provided by Richard J. Pease (Leeds Institute for Cardiovascular and Metabolic Medicine, University of Leeds, Leeds, United Kingdom) and were bred in house at the Seattle Children’s Research Institute vivarium, as needed. Other mouse strains, such as *Cpa3-Cre*; *F13a1^fl/fl^*, and *Lys-Cre*; *F13a1^fl/fl^*, were bred in house at the Seattle Children’s Research Institute vivarium as needed. Controls included WT B6 mice or heterozygous mice that either lacked the Cre recombinase or were heterozygous for the flox (*fl/+)* allele of FXIII.

### Gonadectomies.

GDX mice were obtained using methods described previously (69). Briefly, under deep surgical anesthesia, 7- to 9-week-old male mice were castrated via a single midscrotal incision, and the testes were removed using a cautery pen (Gemini Cautery System, Braintree Scientific Inc.). Hemostasis was verified, and the skin incision was sutured closed. In female mice, 2 flank incisions were performed, and the ovaries were located and exteriorized through the muscle wall. Ovaries were removed using a cautery pen, and hemostasis was verified before suturing both the muscle and skin layers closed. For sham surgery, the same procedures were performed without the removal of the testes or ovaries. Ketoprofen (Zoetis) analgesia was given at the onset of the procedure and continued for the following 2 days. GDX mice were allowed to recover and used as indicated in experiments 1 to 2 weeks after surgical procedures.

### Murine model of GBS infection.

To achieve GBS systemic infection, 9- to 12-week-old mice were injected i.p. with 0.5 to 1 × 10^8^ CFU of GBS. Blood, peritoneal fluid, spleen, and lungs were collected aseptically after 24 hours. Lungs and spleen were homogenized in sterile PBS, and bacterial counts in all samples were determined by plating serial 10-fold dilutions on TSA as described (15, 16). Tridegin (6 μg/mouse in 100 μL PBS) or FXIIIA (1 unit [~20 μg] mouse in 100 μL PBS) was administered to the mice i.v. via the retroorbital route 2 hours after GBS infection. For i.v. GBS inoculation, 9- to 12-week-old mice were injected with 0.5 × 10^8^ CFU of GBS via retroorbital injection, and blood, peritoneal fluid, and spleens were collected aseptically after 24 hours for bacterial enumeration.

### Immunohistochemistry.

Mice were euthanized at 24 hours after GBS or PBS inoculation, and tissues were excised and immediately fixed in 10% formalin overnight at 4°C. Then formalin was replaced with 70% ethanol and stored at 4°C until sectioning. Tissues were sectioned and slides were deparaffinized in Leica Bond Dewax Solution on Leica Bond Automated Immunostainer and rehydrated through 100% ethanol. Antigens were retrieved with citrate buffer (pH 6.0, Leica Bond Epitope Retrieval Solution 1) and with EDTA buffer (pH 9.0, Leica Bond Epitope Retrieval Solution 2) at 100°C for 20 minutes for FXIIIA and GBS staining, respectively. All subsequent steps were performed at room temperature.

After blocking endogenous peroxidase activity with 3.0% H_2_O_2_ for 5 minutes and blocking with 10% normal goat serum in TBS for 10 minutes, the sections were stained with a 1:1,000 dilution of a rabbit monoclonal antibody to mouse FXIIIA (clone EP3372, Abcam) or a 1:400 dilution of a rabbit polyclonal antibody to GBS (catalog ab53584, Abcam) in Bond Primary Antibody Diluent (Leica) for 30 minutes. A peroxidase-conjugated goat polyclonal anti-rabbit IgG secondary antibody (catalog DS9800, Leica) was then applied for 8 minutes. Antibody complexes were visualized using Leica Bond Mixed Refine Substrate Detection (3,3′-diaminobenzidine [DAB]) for 10 minutes. After washing with H_2_O, sections were counterstained with hematoxylin (Leica Bond Refine Kit) for 4 minutes, dehydrated through 100% ethanol, cleared in xylene, and mounted with synthetic resin mounting medium and a 1.5 coverslip. Whole slides were scanned and viewed using the Hamamatsu Nanozoomer Digital Pathology System and evaluated qualitatively by a board-certified veterinary pathologist. Images were captured at ×20 magnification from scanned slides and plated using Adobe Photoshop CC 2015.

### Bleeding time test.

Tail tip bleeding was performed as described previously (17, 32). Briefly, approximately 3 to 4 mm of mouse tail was resected from the tip. Time until bleeding cessation was monitored by blood aspiration onto pieces of filter paper.

### Flow cytometry.

For flow cytometry, see Supplemental Methods.

### Generation of recombinant ScpB expression plasmid.

To facilitate purification of ScpB in *E*. *coli*, an N-terminally glutathione S-transferase–tagged (GST-tagged) ScpB variant was constructed. The coding region of *scpB* was amplified using high-fidelity PCR (Q5 High Fidelity) with GBS COH1 genomic DNA template and primers 5′-GCGAATTCGCGTGACAGAAGACAC-3′ and 5′-GCCTCGAGTGCGTTTTTAGTTTCTTTT-3′. The primers contained *Eco*RI and *Xho*I restriction sites for directional cloning of *scpB* into the GST-expression vector pGEX4T3, creating an in-frame insertion of a GST tag at the N-terminus. The resulting plasmid, pGEX-ScpB, was verified through DNA sequencing and transformed into *E*. *coli* BL21(DE3) pLysS (Invitrogen).

### Purification of recombinant ScpB protein.

*E*. *coli* BL21(DE3) pLysS containing pGEX-ScpB was grown at 37°C in LB media supplemented with ampicillin. At OD_600_ of 0.7, isopropyl-b-d-thiogalactopyranoside (IPTG) was added at a final concentration of 0.2 mM and incubated in a shaking incubator at 30°C for 2 hours. Induced cultures were pelleted and resuspended in BugBuster Protein Extraction Reagent (EMD Millipore Corp) and lysed by gentle mixing of the cell suspension on a rotating platform at room temperature. GST-ScpB was purified from clarified lysates under native conditions by affinity chromatography using Pierce Glutathione Agarose (Thermo Scientific) according to the manufacturer’s instructions, and purified GST-ScpB was reconstituted in PBS and stored in aliquots at –80°C until required for use. For antibody production, the fusion protein was subjected to enzymatic cleavage and elution using the Thrombin Cleavage Kit (catalog 207000, Abcam), and the elution of ScpB was performed per the manufacturer’s instructions.

### Generation of ScpB antibody.

Antisera against the ScpB protein was generated, purified, and titered by Lampire Biological Laboratories. In brief, male and female New Zealand white rabbits (>3 months of age) were immunized s.c. with 0.5 mg of ScpB (lacking GST tag) emulsified in Freund’s adjuvant, administered in 3 doses on days 0, 21, and 42. Complete Freund’s adjuvant was used for the initial injection, and incomplete Freund’s adjuvant was used for the subsequent booster injections. Production and terminal bleeds were performed on days 50 and 73 after immunization. Affinity purification was performed using ScpB protein-affiGel affinity column, followed by HPLC purification, and ELISA assays confirmed that antibody titers were greater than 10^5^ compared with prebleed values, which were at 0 or not detected.

### Generation of ScpBΔPDF.

For generation of the ScpBΔPDF constructs, inverse PCR was performed with 1 ng of pGEX4T3ScpB (above) or pScpB (pBEC1, ref. 37) as the template, the primers COH1*scpB*ΔPDF-F (5′-AACCCTGCAGGAAATGCGTGTCGAAATTGTAAATGG-3′) and COH1*scpB*ΔPDF-R (5′-TTCGACACGCATTTCCTGCAGGGTTTTGACATGAG-3′), and the QuikChange Site-Directed Mutagenesis Kit (Agilent). The PCR products were digested with *Dpn*I (NEB) and then transformed into *E*. *coli* TOP10, and the resultant clones were screened for the correct 330 bp deletion by PCR and confirmed through DNA sequencing. Plasmids were then introduced into either *E*. *coli* BL21 DE3 or into GBS COH1Δ*scpB* as appropriate.

### Crosslinking reactions.

Crosslinking between fibronectin and ScpB was initiated by addition of 1 U thrombin (MilliporeSigma) to a solution containing 2 μM purified ScpB, 1 μM human fibronectin (MilliporeSigma), and 30 μg/mL human FXIIIA (Haematologic Technologies, Inc.). ScpB-only, fibronectin-only, and FXIIIA-negative controls were included. The crosslinking reactions were carried out at 25°C in TBS, pH 7.4 buffer containing 5 mM CaCl_2_ and terminated at various time intervals by the addition of 2% SDS and 10% β-mercaptoethanol and denaturing at 95°C. Proteins were separated via SDS-PAGE on either a 4%–12% bis-tris gel (Invitrogen) run in MES buffer or 3%–8% tris-acetate gels (Invitrogen) in tris-acetate buffer. To detect high–molecular weight crosslinked complexes, proteins were transferred to a nitrocellulose membrane using an iBlot system (Invitrogen) prior to treatment with Odyssey TBS Blocking Buffer (LI-COR Biosciences) or 5% dry milk in PBS. Western blot analysis used monoclonal anti-fibronectin antibody (1:1,500, clone FBN11, Invitrogen) or polyclonal anti-ScpB antibody (1:8,000) from above. IRDye 680RD-conjugated goat anti-mouse (LI-COR Biosciences, catalog P/N 926-68070) and goat anti-rabbit (LI-COR Biosciences, catalog P/N 926-68071) antibodies (1:20,000) were used for detection on an Odyssey CLx from LI-COR. Sixteen-bit high contrast images were produced using ImageJ software (NIH).

### Mouse plasma isolation.

Blood was collected from uninfected 6- to 8-week-old male or female B6 mice via cardiac puncture at a ratio of 9:1 blood/sodium citrate (3.2% buffered, Medicago). Samples were then centrifuged at 5300*g* at 4°C for 15 minutes. The supernatant was collected for use in GBS entrapment assays.

### GBS entrapment assay.

GBS entrapment within fibrin clots was performed as described (33) with some minor modifications. Briefly, GBS strains were grown to OD_600_ = 0.3 in TSB at 37°C with 5% CO_2_, washed twice, and resuspended to the original volume using sodium citrate buffer (12.9 mM). Approximately 100 μL of either normal or FXIIIA-deficient human plasma (George King Biomedical Inc.) or male and female mouse plasma in Z Serum Clot Activator Tubes (VWR) was incubated with 10^7^ CFU (in 100 μL) of GBS for 30 minutes at 37°C. Then 50 μL (0.5 U) of human thrombin (Aniara Diagnostica) was added to each tube, mixed, and incubated for an additional 30 minutes. Following incubation, the clots were overlayed with 1 mL of 1% of the respective plasma samples. The tubes were gently inverted to mix. The supernatant was then removed, and the clots were washed twice and overlayed with 1 mL sodium citrate buffer prior to homogenization. Serial dilutions of the supernatant, the homogenized clot as well as the initial inoculum, were plated on TSA for bacterial enumeration. For estimation of GBS released into the supernatant over time, clots obtained from normal or FXIIIA-deficient plasma with GBS as described above were washed briefly and overlayed with 500 μL of sodium citrate. Samples were incubated at 37°C for 2 hours. Aliquots of the supernatant were then diluted and plated on TSA for bacterial enumeration. GBS entrapment in fibronectin-deficient plasma (Innovative Research) was performed as described above in the presence of 2 mg human fibrinogen (MilliporeSigma) without which clotting was not observed. Normal plasma controls for these experiments also included 2 mg fibrinogen.

For scanning electron microscopy, clots were fixed in one-half strength Karnovsky’s fixative (2.5% glutaraldehyde, 2% paraformaldehyde in 0.1M sodium cacodylate buffer, pH 7.3) overnight at 4°C. Samples were then rinsed with 0.1M cacodylate buffer and dehydrated through a graded series of alcohols and critical point dried (Autosamdri, Tousimis Corp.). Samples were then mounted on stubs and sputter coated with gold/palladium (Denton Desk IV). Images were acquired on a JSM 6610 LV scanning electron microscope at 5 kV and at a working distance of 12 mm (JEOL).

### Incorporation of biotin cadaverine into GBS surface.

These experiments were performed using methods previously described (23). Briefly, GBS strains were grown overnight in TSB to approximately 2 × 10^9^ CFU/mL. Then 50 μL of GBS was added to plasma obtained from either WT C57BL/6J or FXIIIA-deficient mice and incubated at 37°C for 60 seconds. Reactions were initiated by adding 100 μL of thrombin (MilliporeSigma), and 1 mM of the peptide H-1998 (H-Gly-Pro-Arg-Pro-NH_2_) (Thermo Fisher) was added to prevent clotting. Human plasma samples were treated similarly, but without the incubation at 37°C, and the peptide H-1998 was added prior to thrombin. Then 5 mM of biotin cadaverine (Thermo Fisher) was added and samples were incubated for 1.5 hours, rotating at 37°C. Samples were then centrifuged at 6000*g* and washed 3 times with PBS. Then 5 μg/mL of streptavidin-Cy3 (MilliporeSigma) was added and the samples were incubated for 1 hour, rotating at room temperature. After washing twice with PBS, the preparations were fixed with 4% paraformaldehyde at room temperature for 15 to 20 minutes, mounted using antifade diamond mounting media, and left overnight for drying. Control samples were prepared as above, but without the addition of biotin-cadaverine. Images were acquired on a Leica TCS SP5 confocal inverted-base microscope (Leica Microsystems) with a ×63 oil objective. Images were further processed using Leica Application Suite X (version 3.4.2.18368, Leica Microsystems) and ImageJ.

### FXIIIA Western blot.

BMCMCs were isolated and cultured as described previously (16). Cells were pelleted, and proteins were extracted in M-PER lysis buffer supplemented with a protease inhibitor cocktail (Thermo Fisher) on ice for 10 minutes as described (73). Protein content of cell lysates was determined by BCA assay, and 12 μg protein was resolved on NuPAGE Bolt Bis-Tris 4 to 12% gels (Fisher Scientific). Proteins were then transferred to a nitrocellulose membrane, and the blots were probed with the following antibodies: mouse anti-FXIIIa (1:1,000, catalog AC-1A1, Abcam) or control rabbit anti–β-actin (1:5,000, clone 13E5, Cell Signaling Technology), followed by 3 × 5 minutes washes with TBS-T buffer. Secondary antibodies used were IRDye 800CW-conjugated goat anti-mouse IgG (1:10,000, LI-COR Biosciences, catalog P/N: 926-32210) or IRDye 680LT-conjugated goat anti-rabbit IgG (1:10,000, LI-COR Biosciences, catalog P/N: 926-68021) for 30 minutes. Western blots were imaged on a LI-COR Odyssey CLx (LI-COR Biosciences), and densitometry analysis was performed using Image J.

### Statistics.

All experiments represent biological replicates. Two-tailed Mann-Whitney *U* test, 2-tailed Student’s *t* test, Kruskal-Wallis test with Dunn’s multiple-comparison test, or Tukey’s or Šidák’s multiple-comparison test following 1-way or 2-way ANOVA was used to estimate differences as appropriate (see figure legends for details). *P* < 0.05 was considered significant. These tests were performed using GraphPad Prism, (version 9.2.0, for macOS or Windows, GraphPad Software).

### Study approval.

Animal experiments performed were reviewed and approved by the Seattle Children’s Research Institutional Animal Care and Use Committee (protocols 00036 and 00020) or the Michigan State University’s Institutional Animal Care and Use Committee (protocol 02/18-023-01) and performed in strict accordance with the recommendations in the NIH’s *Guide for the Care and Use of Laboratory Animals* (National Academies Press, 2011).

## Author contributions

AMP, KS, PQ, AB, AO, SSS, NBS, RS, CG, AJM, and LR designed the experiments. AMP, KS, PQ, AB, SN, AO, SSS, NBS, RS, YPT, EM, GB, CG, AF, AJS, EG, IM, MC, ES, JMS, AJM, and LR performed the experiments and/or analyzed results. DI and CAB provided tridegin. AMP, KS, AB, NBS, RS, DI, JMS, AJM, and LR wrote the manuscript. All authors reviewed the final version of the manuscript.

## Supplementary Material

Supplemental data

## Version 1 08/25/2022

In-Press Preview

## Version 2 10/17/2022

Electronic publication
