# Supplementary material for: Mast cell–derived factor XIIIA contributes to sexual dimorphic defense against group B streptococcal infections
Source: J Clin Invest. 2022 Oct 17;132(20):e157999. doi: 10.1172/JCI157999 (PMC9566924; doi:10.1172/JCI157999)
Supplement: Supplemental data [file jci-132-157999-s075.pdf]

SUPPLEMENTARY FIGURES

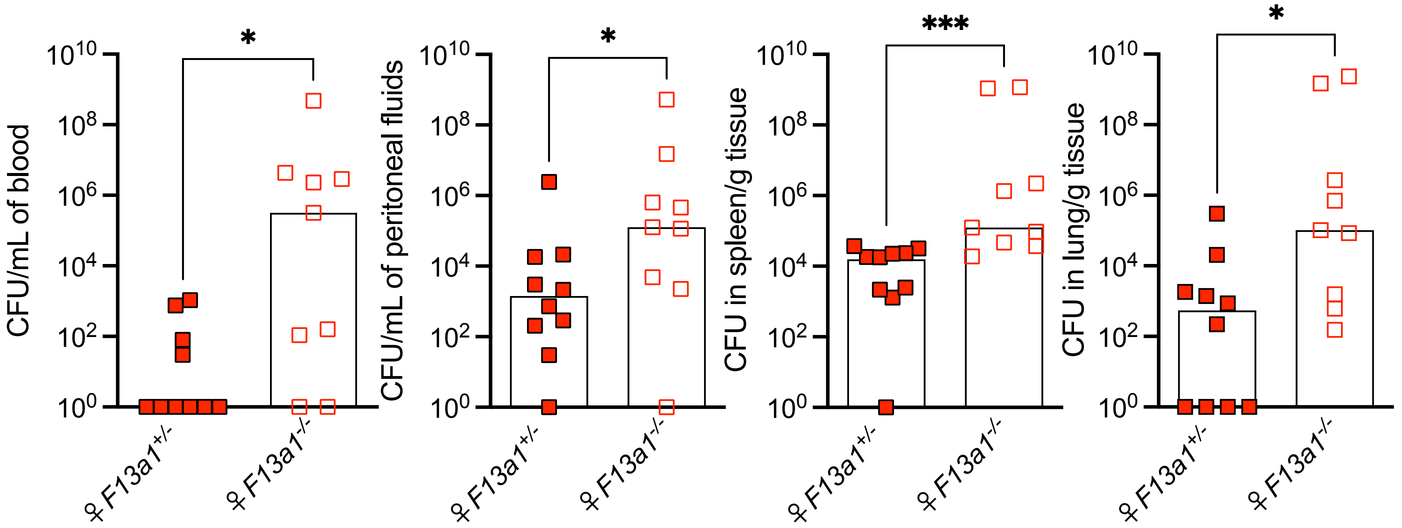

Figure S1. FXIIIA-deficient female mice exhibit increased GBS dissemination during systemic infection with WT GBS strain COH1.

Female FXIIIA-sufficient (*F13a1*$^{+/-}$) and FXIIIA-deficient (*F13a1*$^{-/-}$) mice were infected intraperitoneally (*i.p.)* with 0.5 - 1 X 10^8 CFU of WT GBS strain COH1, *n* = 9-10 mice /group. At 24 h post GBS infection, bacterial burden was evaluated in blood, peritoneal fluids, spleen, and lungs. Data are shown as medians with boxes representing values from individual mice. The Mann-Whitney test was used for comparisons of bacterial burden between FXIIIA-sufficient and FXIIIA-deficient mice. * P <0.05, ***P < 0.001.

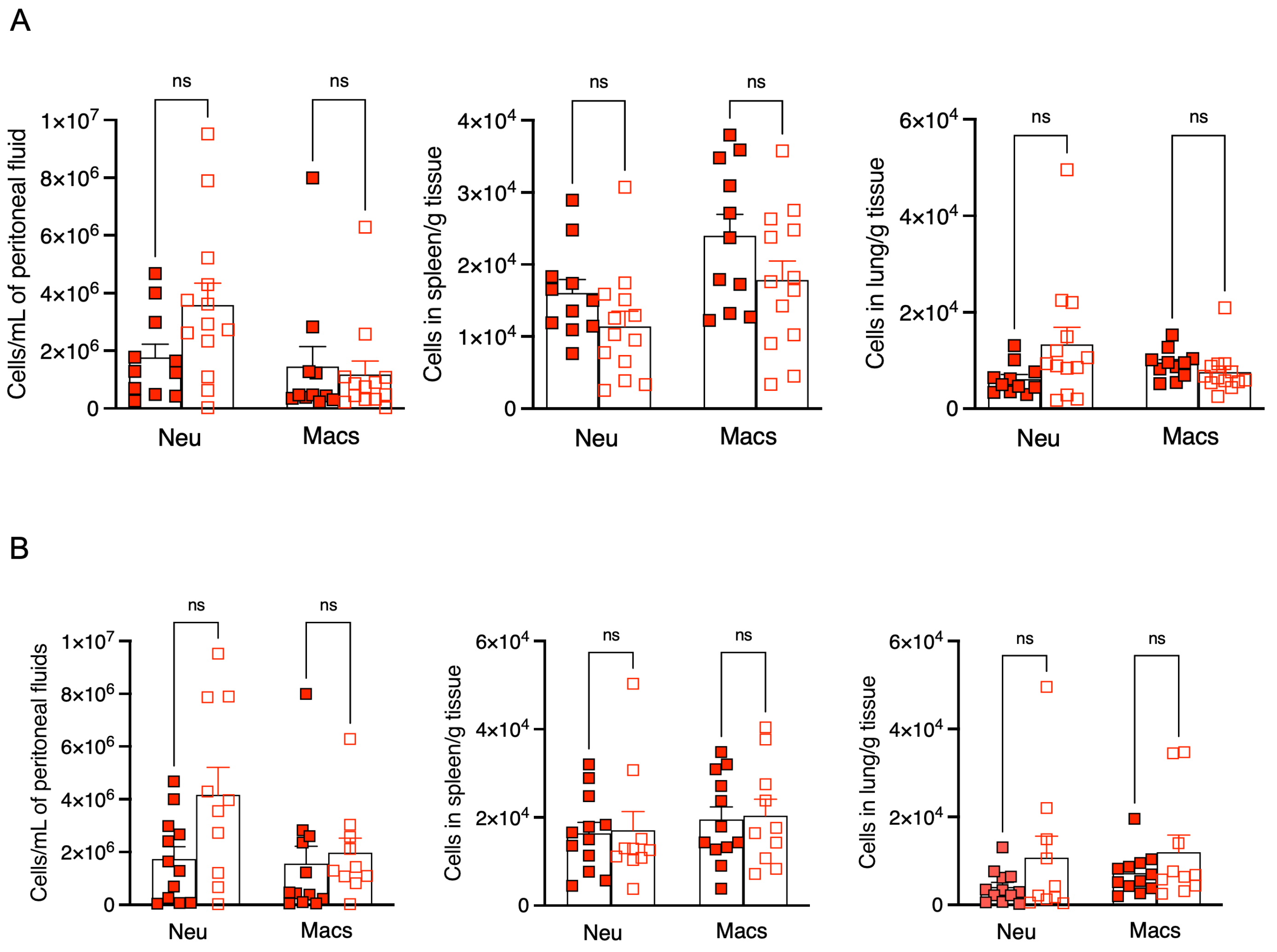

Figure S2. FXIIIA-deficiency does not affect neutrophil and macrophage recruitment during systemic infection with GBS

Female FXIIIA-sufficient (*F13a1*$^{+/-}$, solid boxes) and FXIIIA-deficient (*F13a1*$^{-/-}$, empty boxes) mice were infected intraperitoneally (*i.p.*) with 0.5 - 1 X 10^8 CFU of WT GBS; strain A909 was used for results shown in panel A, *n* = 11-13 mice/group, and strain COH1 was used in panel B, *n* = 10 -12 mice /group. At 24 h post GBS infection, the number of neutrophils and macrophages in the peritoneal, spleen and lung among live cell population was analyzed by flow cytometry (Gr-1$^+$ CD11b$^+$ cells for neutrophils (neu) and F4/80$^+$ CD11b$^+$ for macrophages (macs)). Data are shown as medians with boxes representing values from individual mice. The Mann-Whitney test was used for comparisons of cell numbers between FXIIIA-sufficient and FXIIIA-deficient mice. ns = not significant (P > 0.05).

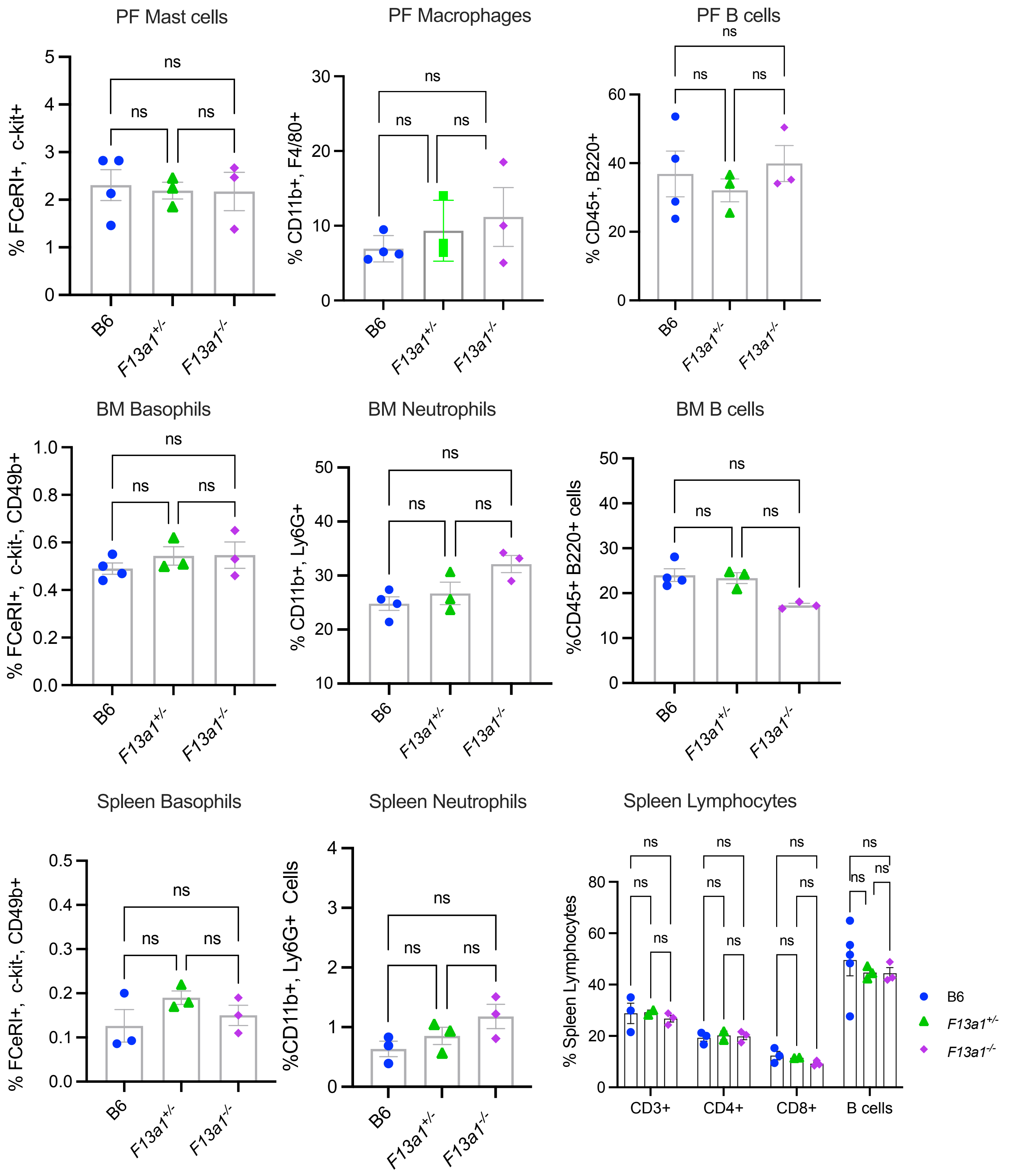

Figure S3. FXIIIA-deficient mice have similar immune cell profiles to FXIIIA-sufficient mice

Bone marrow (BM), peritoneal fluid (PF), and spleen were harvested from 10–12-weeks old C57BL/6 (B6), *F13a1*$^{+/-}$ or *F13a1*$^{-/-}$ mice to assess immune cell populations by flow cytometry. Live cells were gated for markers of basophils (FCƐRI+, c-kit-, CD49b+), mast cells (FCƐRI+, c-kit+), neutrophils (CD11b+, Ly6G+), macrophages (CD11b+, F4/80+), T cells (CD4+ or CD8+) or B cells (CD45+, B220+). Data are shown as the percentage of live cells where each point represents an individual mouse (mean ± SEM). The Kruskal-Wallis test with Dunn's multiple comparison test was used, ns = not significant (P > 0.05).

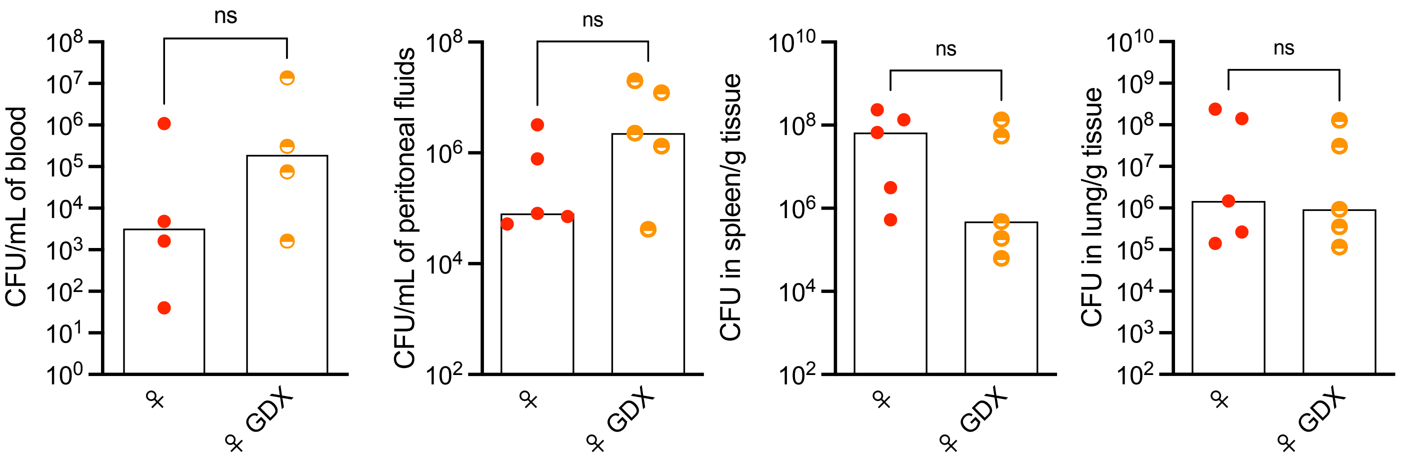

Figure S4. Female sex hormones do not influence susceptibility to GBS systemic infection

Gonadectomized (GDX) or control (sham operated) female mice (*n* = 4-5/group) were infected intraperitoneally (*i.p.*) with 0.5 - 1 X 10^8 CFU of WT GBS A909 and bacterial burden was evaluated in blood, peritoneal fluids, spleen, and lungs. The Mann-Whitney test was used for comparisons of bacterial burden between mice. Data are shown as medians with circles representing values from individual mice, ns: not significant (P > 0.05).

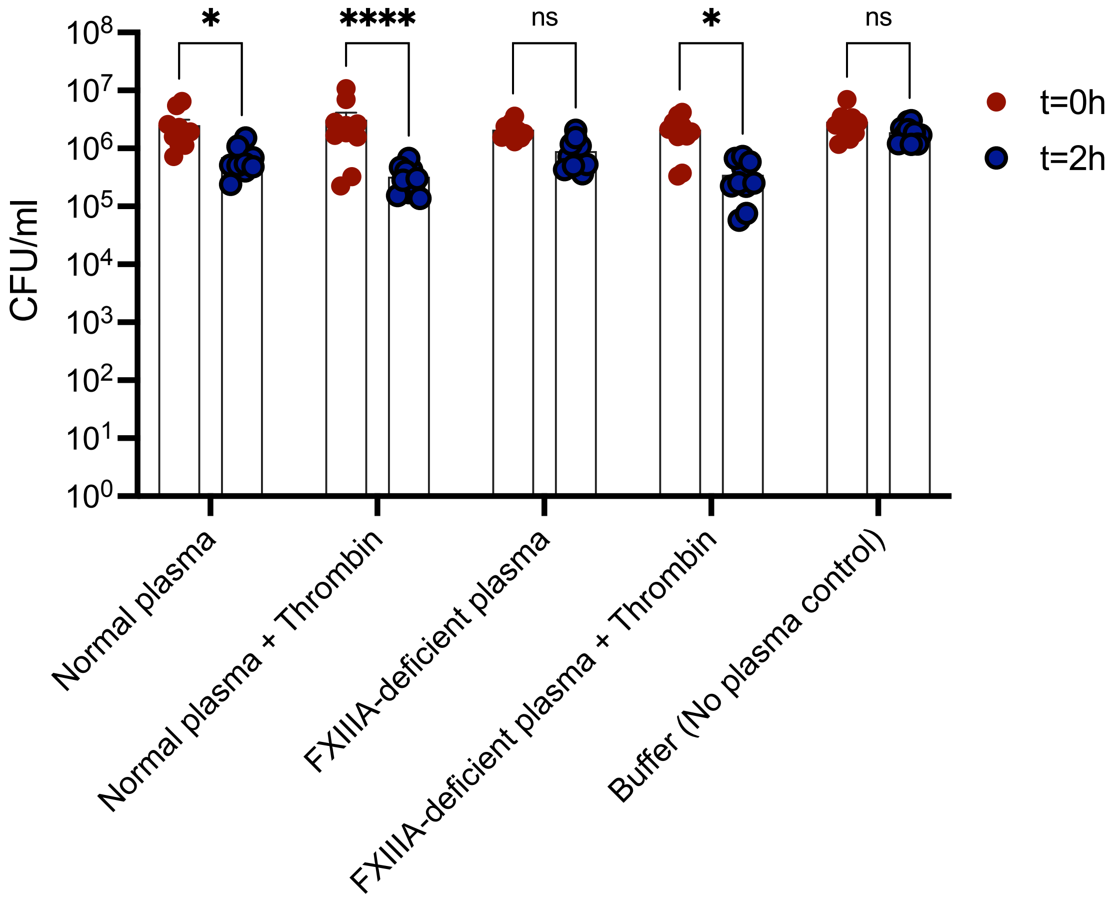

Figure S5. GBS exhibit diminished survival in thrombin activated plasma

The GBS WT strain COH-1 was incubated in normal or FXIII-deficient human plasma (diluted 1:100) with and without thrombin. At the indicated time points *i.e.,* t = 0 and t = 2 hrs, GBS CFU were enumerated by plating serial dilutions onto TSB agar. Controls included GBS incubated in buffer only (sodium citrate). The data represent the means and SEM of five independent experiments. Sidak's multiple comparison test following 2-way ANOVA was used for comparisons between groups, * P <.01, ****P <.0001, ns = not significant.

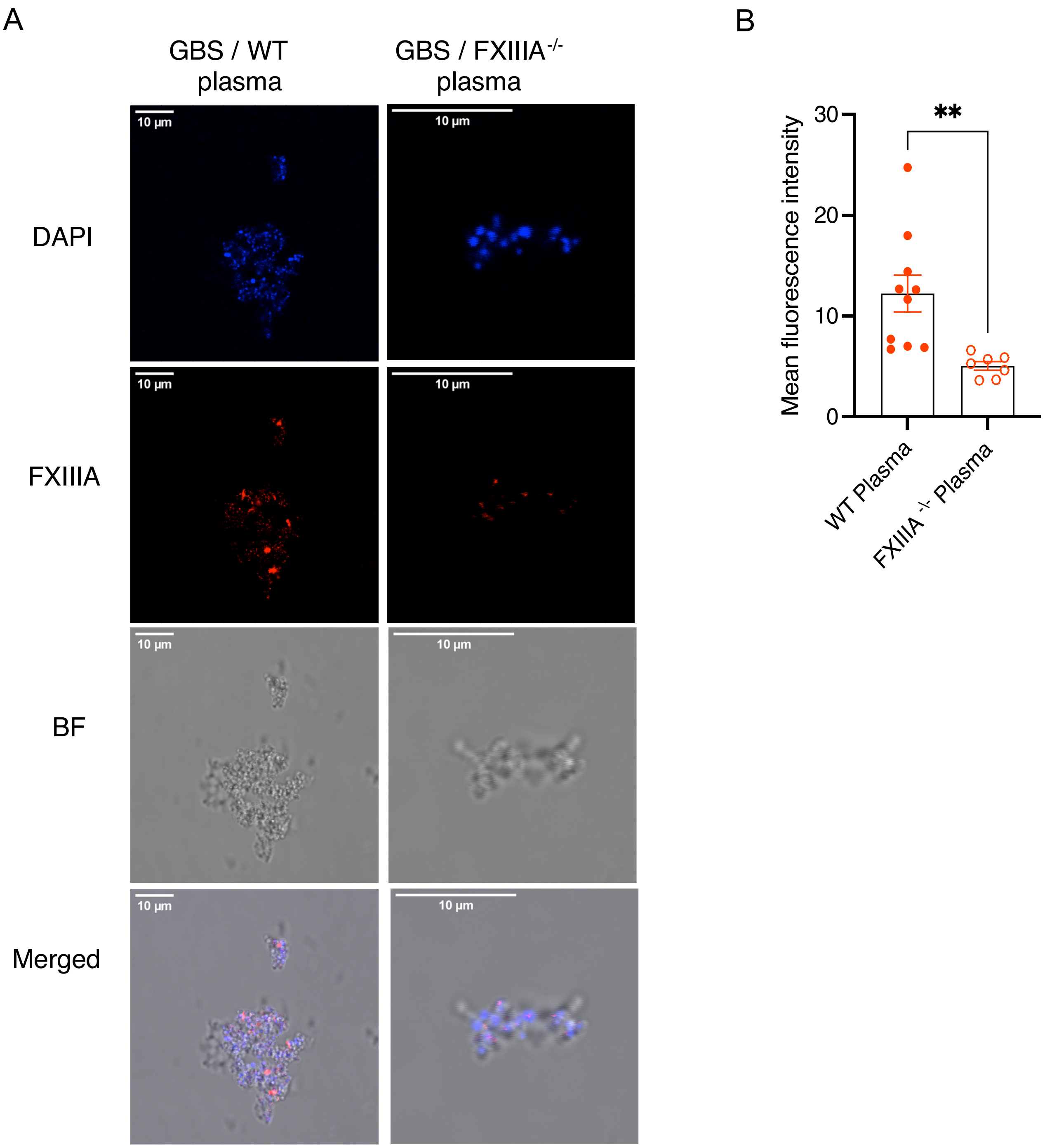

Figure S6. FXIIIA transglutaminase activity on GBS cell surface in the presence of mouse plasma

(A, B) Plasma obtained from WT or FXIIIA-deficient mice (*F13a1*$^{-/-}$) was activated with thrombin in the presence of WT GBS strain COH1. Biotin-cadaverine and Streptavidin-cy3 were used to visualize FXIIIA transglutaminase activity at the bacterial surface using immunofluorescence/confocal microscopy. DAPI and bright field images are also shown. (B) Quantification of FXIIIA transglutaminase activity via immunostaining fluorescence intensity. $^{**}P < 0.01$, 2-sided, unpaired t test; data represent the mean with error bars ± SEM.

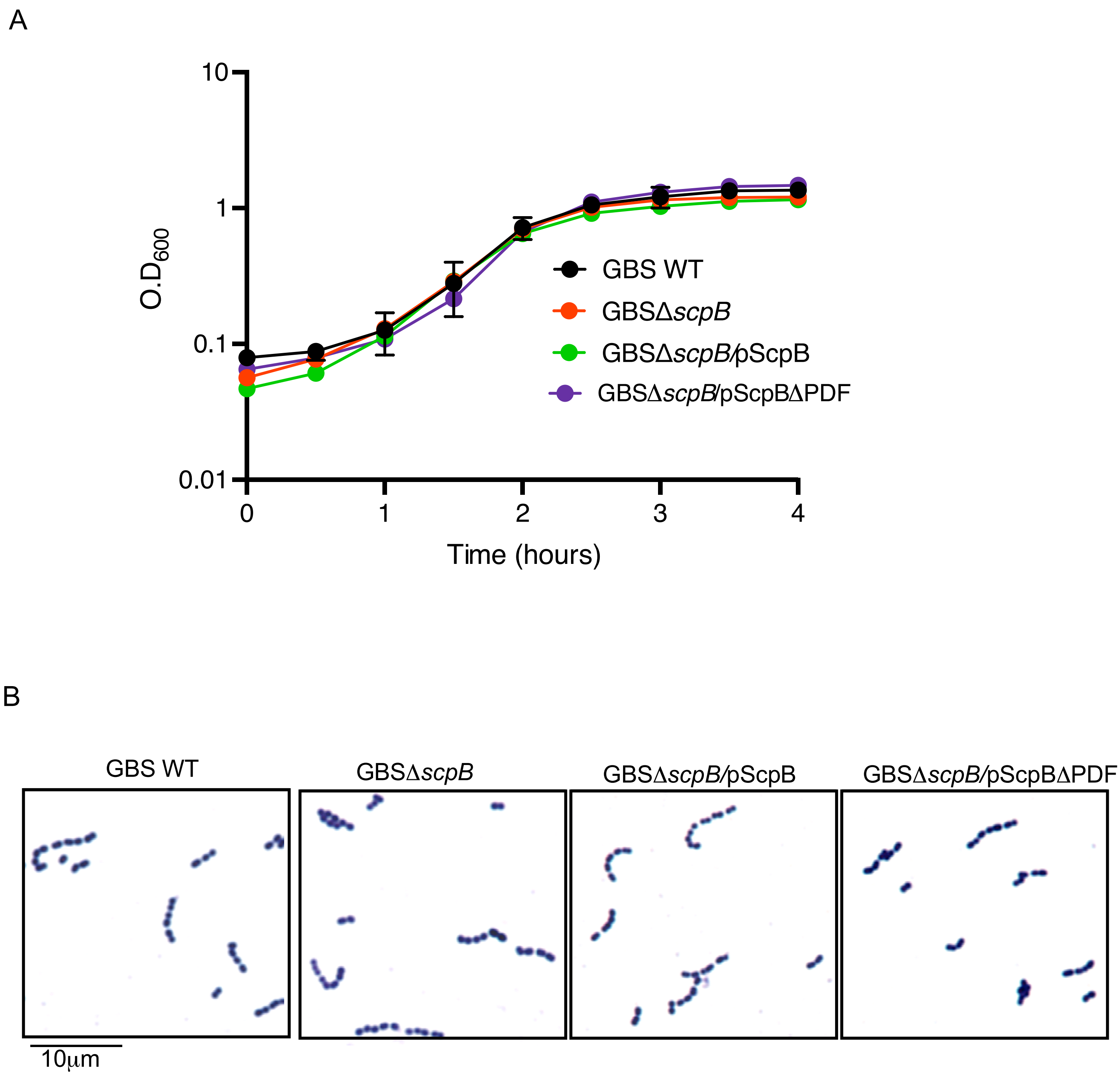

Figure S7. ScpB proficient and deficient GBS strains do not exhibit significant differences in growth rate or colony morphology

(A) Overnight cultures of GBS strains (WT, Δ*scpB* mutant and the ScpB complemented strain with and without the PDF region) derived from COH1 were sub-cultured 1:20 in TSB and incubated at 37°C with 5% CO_2. The optical density at 600nm ($O.D._{600}$) was measured every 30 minutes until stationary phase ($O.D._{600}$= 1.0) was reached. (B) Approx. 50uL of overnight GBS cultures were fixed on glass slides and gram staining was performed using the Gram Stain Kit (Epredia ™) following manufacturer's instructions. Slides were imaged using a microscope (Keyence Bz-X710).

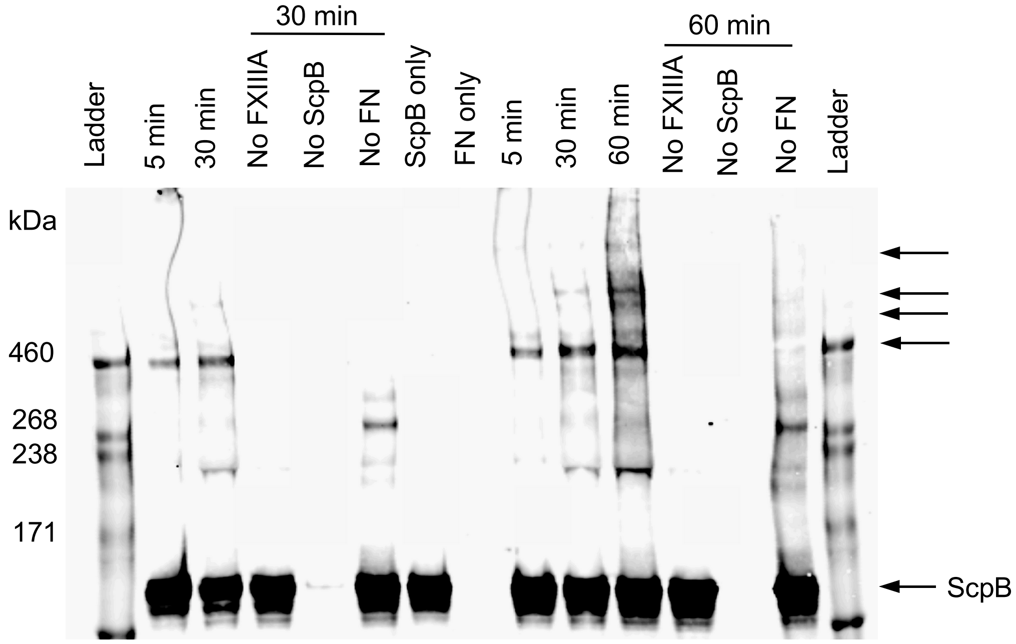

Figure S8. Factor XIIIA-catalyzed crosslinking between fibronectin (FN) and purified ScpB.

ScpB (2 µM) were incubated with fibronectin (FN, 1 µM) and Factor XIIIA (30 µg/ml) in TBS, pH 7.4, 5 mM $CaCl_2$ buffer at 25°C from 0 - 60 minutes. Aliquots were removed at the indicated times and were separated on SDS-PAGE using 3-8% tris-acetate gels (Invitrogen) in tris-acetate buffer followed by western blotting using the anti-ScpB antibody. Following iBlot transfer, blocking was performed using 5% dry milk in PBS. Controls included reactions lacking either FXIIIA or ScpB or, reactions containing only FN or ScpB. Arrows shown indicate the positions of ScpB and products of the crosslinking reaction.

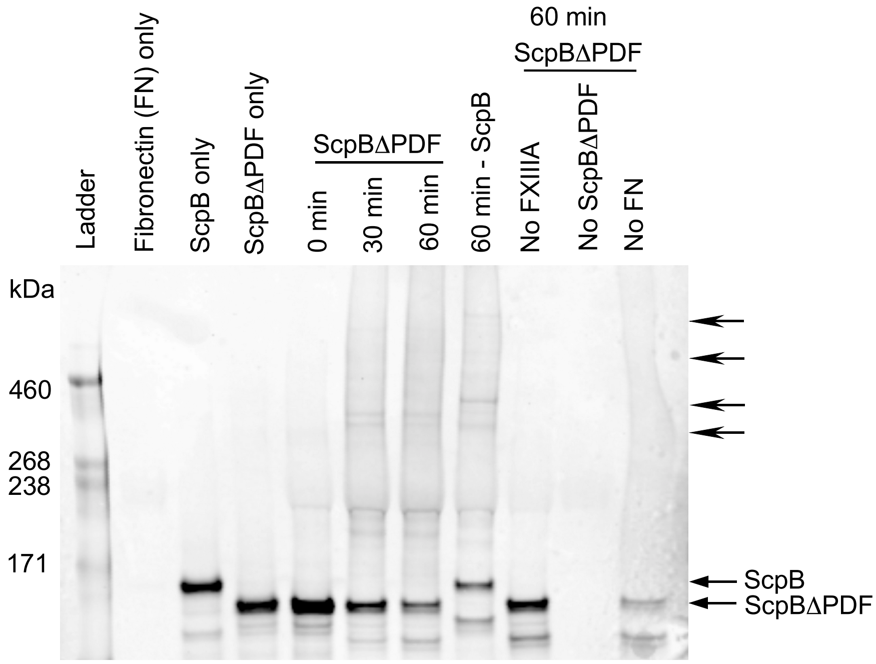

Figure S9. Factor XIIIA-catalyzed crosslinking between fibronectin (FN) and ScpB lacking the fibronectin binding PDF domain.

ScpBΔPDF or ScpB (~2 μM) were incubated with fibronectin (1 μM) and Factor XIIIA (30 μg/ml) in TBS, pH 7.4, 5 mM $CaCl_2$ buffer at 25°C from 0 - 60 minutes. Aliquots were removed at the indicated times and were separated on SDS-PAGE followed by western blotting using the anti-ScpB antibody. Controls included reactions lacking either FXIIIA, ScpB, or reactions containing only Fibronectin or ScpB. Controls included fibronectin (FN) only, ScpB only, SpcBΔPDF only, a 60 min reaction with full length ScpB as well as 60 min reactions with ScpBΔPDF but lacking either FXIIIA, ScpBΔPDF, or FN Arrows shown indicate the positions of the ScpB, ScpBΔPDF and products of the crosslinking reaction.

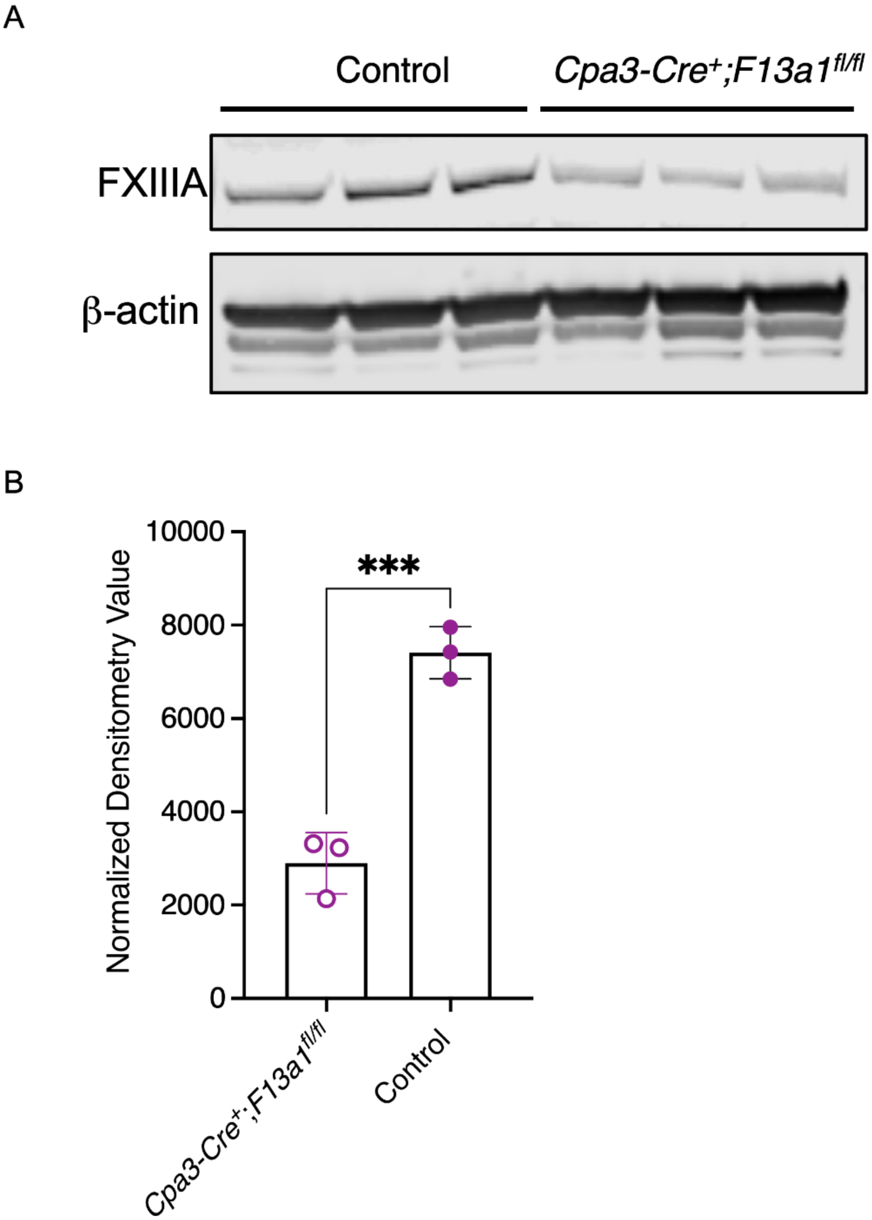

Figure S10. Decreased FXIIIA levels in conditional mast cell knock out mice

Western blots were performed on equivalent amounts of protein isolated from bone marrow derived mast cell (BMCMC) from either *Cpa-cre3*$^{+}$;*F13a1*$^{fl/fl}$ mice or controls. (A) Immunoblots were performed on BMCMC lysates with FXIIIA antibody or control β-actin antibody, respectively. (B) Densitometric quantification of FXIIIA normalized to β -actin. Data shows mean ± SD from three independent samples. Unpaired t-test, *** $P < 0.001$.

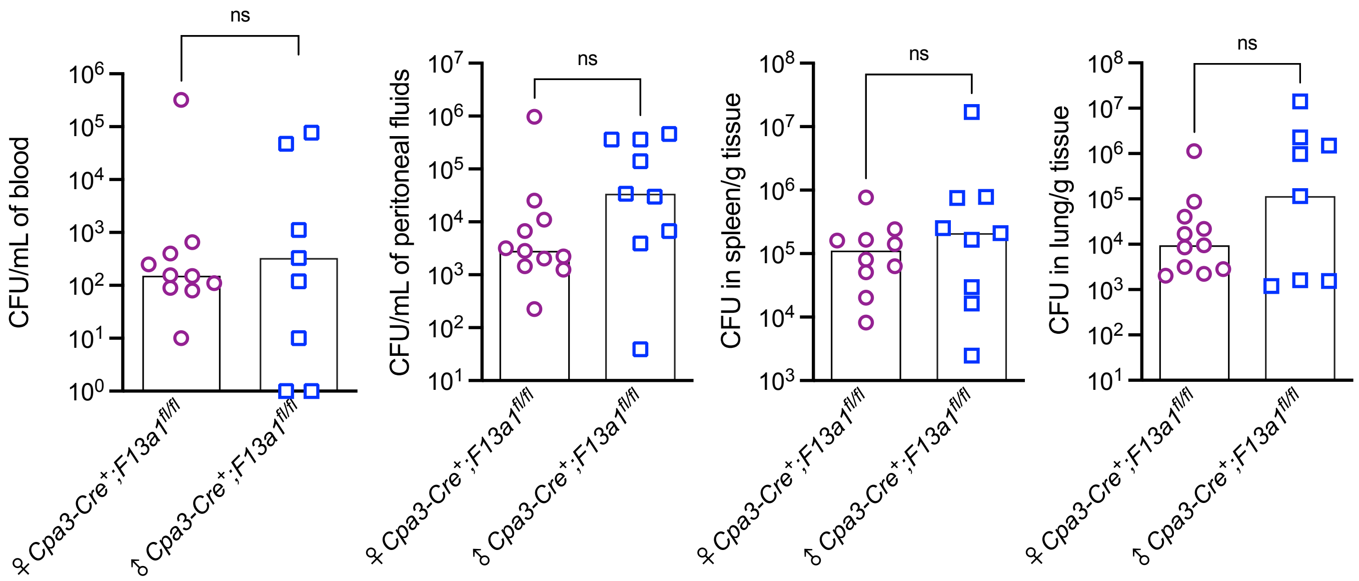

Figure S11. Male and female mice lacking mast cell derived FXIIIA exhibit similar susceptibility to GBS infections

Bacterial burden was evaluated in blood, peritoneal fluids, spleen, and lungs at 24 hours post *i.p.* injection of GBS (0.5 - 1 X 10^8 CFU of WT A909) in male and female mice deficient in expression of FXIIIA in mast cells (*Cpa-cre3*$^+$; *F13a1*$^{fl/fl}$) (*n* = 9 -11/group). Data are shown as medians with boxes or circles representing values from individual mice. The Mann-Whitney test was used for comparisons of bacterial burden between the GBS strains. ns= not significant, $P > 0.05$.

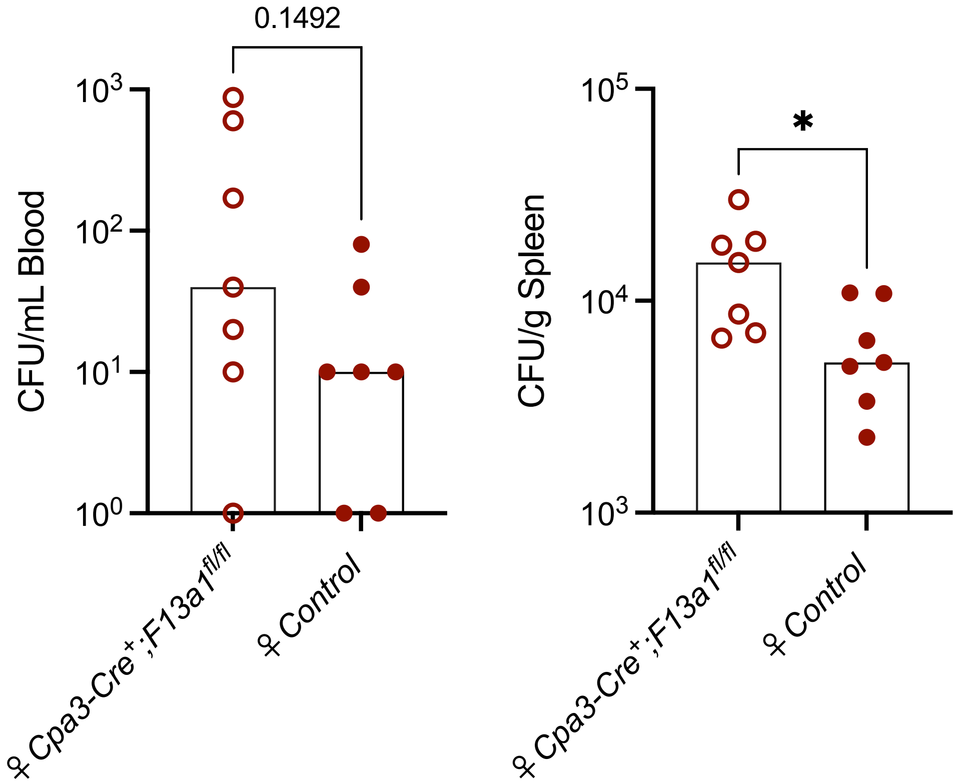

Figure S12. Female mice lacking mast cell derived FXIIIA exhibit increased dissemination after intravenous GBS inoculation

Bacterial burden was evaluated in blood, spleen and peritoneal fluids at 24 hours post intravenous (*i.v.*) injection of GBS (0.5 X 10^8 CFU of WT A909) in female mice deficient in expression of FXIIIA in mast cells (*Cpa-cre3*$^+$; *F13a1*$^{fl/fl}$) or respective controls (*n* = 7/group). Of note, bacteria were not recovered in the peritoneal fluids from either mouse strain from this route of GBS infection. Data are shown as medians with circles representing values from individual mice. The Mann-Whitney test was used for comparisons of bacterial burden between the GBS strains. * $P < 0.05$.

Flow cytometry methods

Single cell suspensions for bone marrow were generated by flushing the tibia and femur bones from one leg using a 22-gauge needle and PBS with 2% heat-inactivated fetal bovine serum (HI-FBS). The marrow was gently disrupted using a 22-gauge needle and red blood cells were lysed for 30 seconds using a 0.2% sodium chloride solution followed by neutralization with a 1.6% sodium chloride solution. After a wash with PBS, cells were resuspended in PBS + 2% HI-FBS and left on ice until staining for flow cytometry. Peritoneal fluid was collected after flushing the peritoneal cavity with 8 mL of PBS + 2% HI-FBS. Spleen single cell suspensions were generated by gently disrupting the spleen between two frosted microscope slides with 2 mL of PBS + 2% HI-FBS. Red blood cells were lysed for 30 seconds with ACK lysing buffer. After a wash with PBS, cells were resuspended in PBS + 2% HI-FBS and left on ice until staining for flow cytometry and stained with a combination of the following antibodies: Alexa Fluor 700-conjugated mAb to Ly-6G/Ly-6C (clone RB6-8C5, catalog number 108421), allophycocyanin (APC)-conjugated mAb to F4/80 (clone BM8, catalog number 123115), and eFluor 450-conjugated mAb to CD11b (clone M1/70, catalog number 48-0112-82). Alexa Fluor 488-conjugated mAb to B220 (clone RA3-6B2, catalog number 103211, BioLegend), APC-conjugated mAb to CD49b (clone DX5, catalog number 108909, BioLegend), APC-conjugated mAb to CD8a (clone 53-6.7, catalog number 100711, BioLegend), APC-Cy7 conjugated mAb to c-kit (clone 2B8, catalog number 105826, BioLegend), BV605 conjugated mAb to Ly6G (clone 1A8, catalog number 127639, BioLegend), FITC conjugated mAb to CD3 (clone 145-2C11, catalog number 35-0031-U100, Tonbo Biosciences), PE conjugated mAb to CD4 (clone GK1.5, catalog number 12-0041-82, eBioscience), PE conjugated mAb to FCƐRIa (clone MAR-1, catalog number 134308, BioLegend), PE conjugated mAb to IgE (clone RME-1, catalog number 406907, BioLegend), and PerCP/Cy5.5 conjugated mAb to CD45 (Clone 30-F11, catalog number 103132, BioLegend). 4', 6-diamidino-2-phenylindole (DAPI) (catalog number D9542, Sigma-Aldrich) was used to detect dead cells. Only cells that were negative for DAPI were used for the cell population analysis. The cells were acquired on a BD LSR II flow cytometer with the FACSDiva software and analyzed with the FlowJo software (version 8.8.7, Tree Star).
